# Decoding Biodiversity in Baiyangdian Lake: A DNA Barcode Reference Library for Aquatic Insects

**DOI:** 10.3390/insects17010060

**Published:** 2026-01-01

**Authors:** Ya-Jun Qiao, Ze-Peng Wang, Meng-Yu Lv, Pei-Dong Su, Tong Wu, Hai-Feng Xu, Yu-Fan Li, Xiao-Long Lin, Chun-Hui Zhang

**Affiliations:** 1 School of Chemical and Environmental Engineering, China University of Mining and Technology (Beijing), Beijing 100083, China; adiu1213@163.com (Y.-J.Q.); 15130208699@163.com (Z.-P.W.); spd1194042797@gmail.com (P.-D.S.); 2 Ecology and Environment Bureau of Xiong’an New Area, Xiong’an 071800, China; 3 Ecological and Environmental Monitoring Center of Xiong’an New Area, Xiong’an 071800, China; mengyulvl@163.com (M.-Y.L.); sthjjwt@163.com (T.W.); 4 Engineering Research Center of Environmental DNA and Ecological Water Health Assessment, Shanghai Ocean University, Shanghai 201306, China; haifengxu2024@gmail.com (H.-F.X.); liyufan916@gmail.com (Y.-F.L.); 5 Shanghai Universities Key Laboratory of Marine Animal Taxonomy and Evolution, Shanghai Ocean University, Shanghai 201306, China

**Keywords:** DNA barcoding, *COI*, species identification

## Abstract

Freshwater ecosystems are among the most vulnerable habitats worldwide, and reliable biodiversity assessment is essential for their conservation. Baiyangdian Lake, the largest freshwater lake in northern China, has undergone severe ecological degradation but is now experiencing recovery through restoration efforts. To provide a molecular basis for monitoring biodiversity, we constructed a *COI* DNA barcode reference library of aquatic insects from Baiyangdian Lake. From January 2023 to May 2025, systematic sampling across representative habitats yielded 315 high-quality sequences covering 104 species, 74 genera, and 33 families within eight insect orders. Diptera, particularly Chironomidae, showed the highest diversity, followed by Odonata. Phylogenetic analysis using maximum likelihood resolved all orders and families as well-supported monophyletic groups, demonstrating strong congruence with morphological taxonomy. Genetic distance analysis revealed a pronounced barcode gap, with mean intraspecific divergence of 0.46% and nearest-neighbor divergence exceeding 15%, confirming the discriminatory power of *COI* for species identification. Accumulation curves indicated that genus-level diversity is largely captured, while species-level diversity, especially among Diptera, remains incompletely revealed. This study provides the first comprehensive DNA barcode reference library for Baiyangdian aquatic insects, supporting ecological restoration evaluation, eDNA applications, and regional biodiversity conservation strategies.

## Simple Summary

Freshwater ecosystems are highly threatened, and reliable biodiversity monitoring is vital for their protection. Baiyangdian Lake, the largest freshwater lake in northern China, has suffered severe degradation but is now being restored. To support these efforts, we created a DNA barcode reference library of aquatic insects using the *COI* gene. Between 2023 and 2025, we collected samples from different habitats and generated 315 DNA sequences representing more than 100 species. The results showed rich insect diversity, with midges and dragonflies as dominant groups, and confirmed that DNA barcoding can clearly distinguish all species. This study provides the first comprehensive genetic reference for Baiyangdian aquatic insects. It will help track biodiversity recovery, guide conservation strategies, and supply valuable data for global databases, offering an efficient tool to evaluate ecological restoration and protect freshwater ecosystems.

## 1. Introduction

Global biodiversity is facing an unprecedented decline, with freshwater ecosystems showing particularly severe degradation [1,2]. According to the Global Assessment Report released by Intergovernmental Science-Policy Platform on Biodiversity and Ecosystem Services (IPBES) in 2019, nearly one million species of plants and animals are at risk of extinction. Freshwater habitats, due to their limited area and strong dependence on hydrological dynamics, have been identified as one of the most vulnerable ecosystem types worldwide [3]. Although freshwater ecosystems cover only about 0.8% of the Earth’s surface, they support more than 10% of known species and provide critical ecosystem services such as drinking water supply, nutrient cycling, fisheries resources, and climate regulation [4,5,6]. However, over the past 50 years, populations of freshwater species have declined by more than 80%, a rate far exceeding that of marine and terrestrial ecosystems [2,7]. This drastic decline has been driven by multiple pressures, including over-exploitation of water resources, pollution, invasive species, and climate change [4,8,9,10].

The North China Plain, characterized by scarce water resources and dense human populations, has long placed enormous pressure on its wetland ecosystems [11]. Baiyangdian Lake, the largest freshwater lake in northern China, is located in the central part of Hebei Province within the Xiong’an New Area. Covering an area of about 366 km^2^, it belongs to the Daqing River system of the Hai River Basin and serves as a crucial wetland ecosystem in the region [12]. Baiyangdian Lake plays an essential role in water conservation, flood regulation, and climate moderation, while also providing habitat for numerous waterbirds and aquatic organisms [13]. However, due to reduced inflow from upstream, agricultural source pollution, land reclamation, and intensive human disturbances, the ecosystem of Baiyangdian Lake suffered severe degradation over past decades [14,15,16,17]. Water quality once deteriorated to below Class V, wetland area shrank drastically, biodiversity declined sharply, and communities of fish and benthic invertebrates became homogenized, leading to significant loss of ecosystem functions [14,16,18,19].

Since the establishment of the Xiong’an New Area in 2017, a series of systematic ecological restoration projects have been implemented in Baiyangdian Lake. These include pollution control, ecological water replenishment, wetland vegetation restoration, optimization of aquatic plant community structure, and stock enhancement of aquatic organisms [20]. As a result of these continuous efforts, the water quality of Baiyangdian Lake has improved dramatically from below Class V to a stable Class III, placing it among the lakes with good water quality nationwide [21]. Ecological restoration has not only improved the aquatic environment but also created opportunities for the return of typical freshwater invertebrates [22]. In April 2024, based on morphological screening combined with *COI* DNA barcode comparison (sequence similarity reaching 99%), the mayfly *Baetis majus* was recorded for the first time in Baiyangdian Lake (previously known from the Russian Far East), marking the first record of this species in China [23,24]. The discovery of its larvae in Baiyangdian not only enriches the faunal records of Ephemeroptera in China but also indicates significant improvement in local water quality and ecological conditions. This finding highlights the potential of DNA barcoding for ecological restoration monitoring and the detection of cryptic species [25].

**Figure 1:**
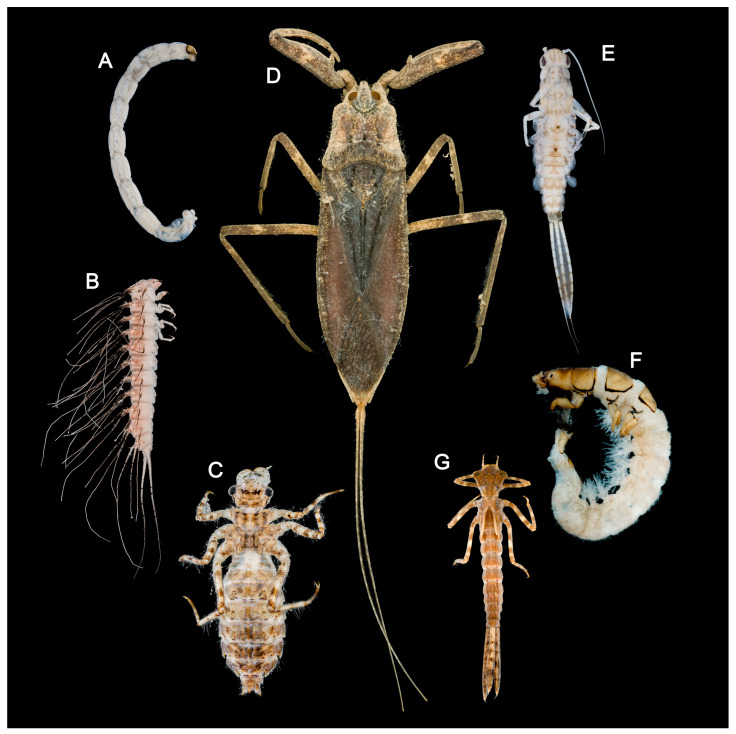
Photos of aquatic insects in Baiyangdian Lake: (**A**) larva of *Chironomus* sp.; (**B**) larva of *Peltodytes* sp.; (**C**) larva of *Deielia phaon*; (**D**) adult of *Laccotrephes japonensis*; (**E**) larva of *Cloeon viridulum*; (**F**) larva of *Cheumatopsyche brevilineata*; (**G**) larva of *Platycnemis phyllopoda*. The picture displays the actual specimen images of the groups collected in this study.

Nevertheless, biodiversity recovery and the reconstruction of ecosystem functions demand higher standards for monitoring technologies [26]. As a representative freshwater habitat, Baiyangdian Lake harbors a large number of small or cryptic aquatic invertebrates (Figure 1), such as Chironomidae, Ephemeroptera and Trichoptera. These groups are not only key components of food webs but also sensitive indicators of ecological health [27]. However, traditional taxonomy based on morphological traits has clear limitations. Many taxa have poorly defined larval characteristics, leading to misidentification or overlooked species. Moreover, morphological identification is time-consuming and requires expert knowledge, making it difficult to meet the needs of large-scale, high-frequency ecological monitoring [28].

DNA barcoding, which uses short standardized gene fragments for rapid and accurate species identification, greatly overcomes these limitations [28]. Its advantages include the ability to identify larvae, fragments, and cryptic species, high throughput potential, and globally standardized data that can be compared across regions [29]. In recent years, the integration of environmental DNA (eDNA) with DNA barcoding has opened new opportunities for freshwater biodiversity monitoring. eDNA technology, which detects genetic material released into the water, enables non-invasive, rapid species detection and is well suited for early warning and large-scale surveys [30]. When eDNA analysis is coupled with high-quality DNA barcode reference libraries, species-level identification can be achieved without collecting many physical specimens in some situations, greatly enhancing the efficiency and sensitivity of ecological restoration assessments [31].

However, one of the major bottlenecks in eDNA applications lies in the lack of reference databases [32,33,34]. Currently, the DNA barcode coverage of freshwater invertebrate groups in Asia (particularly in northern China) is severely inadequate, directly affecting the accuracy of eDNA sequence matching and species identification [35]. Therefore, building high-quality, region-specific local DNA barcode reference databases is a prerequisite for supporting eDNA monitoring [36]. This study aims to integrate traditional taxonomy with molecular methods to construct a comprehensive, accurate, and regionally tailored DNA barcode reference library for the main biological groups in Baiyangdian Lake. Such a resource will promote biodiversity data standardization and sharing, improve species identification accuracy and efficiency, and provide a scientific basis for evaluating ecological restoration and developing conservation strategies in Baiyangdian Lake. At the same time, this work will help fill the gap in freshwater DNA barcode data for Asia, reduce global coverage biases, and provide both a data foundation and theoretical support for promoting international cooperation in freshwater ecosystem conservation.

## 2. Materials and Methods

### 2.1. Sample Collection and Identification

From January 2023 to May 2025, we continuously conducted a systematic survey of aquatic insect diversity in Baiyangdian Lake, China. Sampling was mainly carried out using a D-net at predetermined habitat sites. Collected samples were immediately subjected to preliminary rinsing and sorting in the field and were carefully separated according to their life stages (adult, pupa, larva). The geographic distribution of sampling sites was relatively concentrated, ranging from 38.7480° to 38.9980 °N in latitude, 115.7430° to 116.0960 °E in longitude, and 3–13 m in elevation (Table S1). All samples were collected from surface waters, essentially covering the shallow-water habitats of the Baiyangdian Lake region.

To maximize the preservation of both morphological and molecular integrity, we applied differential preservation strategies: adult specimens were stored in 75% ethanol to prevent excessive brittleness of their chitinous exoskeleton, while pupae and larvae, with softer tissues, were preserved in 95% ethanol to ensure effective dehydration and fixation [37]. All specimens were sealed in screw-cap sample vials with waterproof labels containing detailed information and then placed in light-proof refrigerated storage at 4 °C to slow down ethanol evaporation and ensure long-term stability.

In the laboratory, identification was conducted under a stereomicroscope for overall morphological observation and measurement, while critical structures requiring dissection (e.g., genitalia, mouthparts) were examined under a compound microscope. Identification strictly followed authoritative taxonomic literature for aquatic insects [38,39,40,41,42,43,44,45,46,47,48]. All voucher specimens and related original samples that have been fully identified are catalogued systematically and permanently preserved as valuable biological resources in the standardized specimen repository of the Ecology and Environment Bureau of Xiong’an New Area.

### 2.2. Molecular Experiments

For each adult sample, one side legs or thoracic muscle was used for DNA extraction; larval samples were dissected to remove the gut, after which thoracic and abdominal muscles were used; pupal samples were directly processed for muscle tissue. DNA extraction was performed using the Universal Genomic DNA Kit (CWBIO, Taizhou, China), strictly following the manufacturer’s protocol. The *COI* barcode region was amplified with universal primers LCO1490 and HCO2198 [49]. PCR reaction systems for Chironomidae followed previous study [50], while the amplification system and thermal cycling program for other insect groups are shown in Table A1 and Table A2. PCR products were purified and then sent to Genewiz Co., Ltd. (Suzhou, China) for Sanger sequencing. All DNA voucher samples generated in this study are currently deposited at the Ecology and Environment Bureau of Xiong’an New Area.

### 2.3. Sequence Processing and DNA Barcode Analysis

This study integrated 315 sequences generated by us into the Barcode of Life Data System (BOLD) [51] under the dataset “DNA barcodes of Aquatic insects from Baiyangdian Lake, Hebei, China (DS-BYDAI)” for subsequent analyses. Raw sequences were assembled and quality-checked in Geneious Prime v2024.0.5 (Biomatters, Auckland, New Zealand) [52], then aligned with MUSCLE v3.8.31 (Edmonds, WA, USA) [53] implemented in MEGA 12 (Philadelphia, PA, USA) [54] to screen for stop codons and translation errors, ensuring sequence reliability.

Phylogenetic analysis was conducted in a neighbor-joining (NJ) framework using MEGA 12. All *COI* barcode sequences were first aligned with MUSCLE before being analyzed. Substitution model was selected with the Kimura 2-parameter (K2P) model (Kyoto, Japan) [55] with 1000 bootstrap replicates and the pairwise deletion method to assess branch support, providing robust evaluation of branch stability. Gaps and ambiguous bases were treated as missing data, and other parameters remained default. The final NJ tree was visualized and edited in FigTree v1.4.4 (Edinburgh, UK) [56].

Further analyses in the BOLD workbench (http://www.boldsystems.org, 31 October 2025) included pairwise genetic distance calculations and DNA barcode gap assessment. Analyses were based on the standard *COI*-5P region (658 bp), with a minimum sequence length threshold of ≥200 bp. Gaps and ambiguous bases were handled using pairwise deletion. Genetic distances were summarized at both genus and species levels, reporting minimum, maximum, mean, and standard error. Barcode gap analysis compared each species’ maximum intraspecific divergence against the minimum nearest-neighbor divergence, visualized as histograms to assess species-level genetic differentiation.

To evaluate sampling completeness and compare diversity accumulation patterns across groups, accumulation curves were generated using the Accumulation Curve function. Analyses were performed at the genus and species levels, with randomized sampling (20 iterations) to minimize order effects. Results were grouped as Multiple Graphs by Taxonomy Family, enabling comparison of sampling efficiency across families. All other parameters remained default.

## 3. Results

### 3.1. Sequence Information

**Figure 2:**
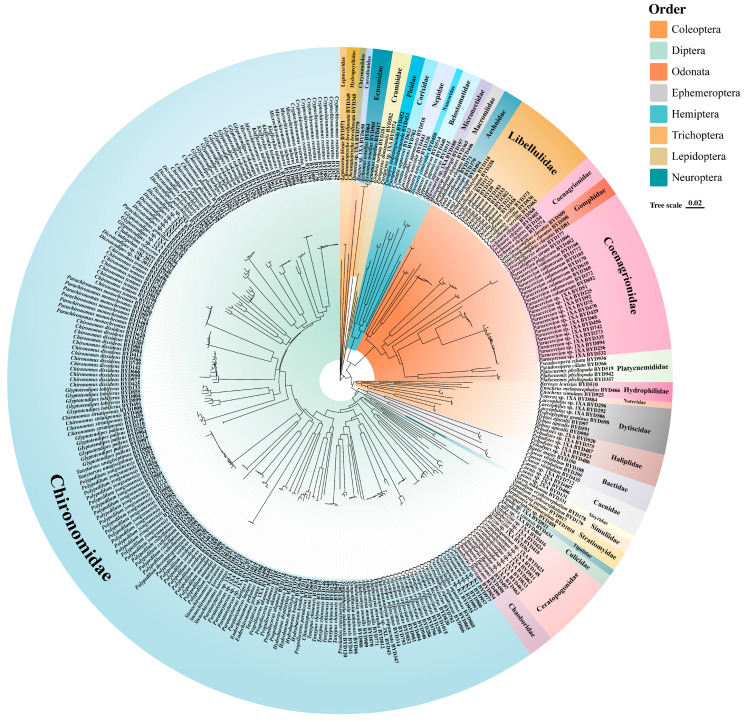
Neighbor-joining tree of aquatic insects from Baiyangdian Lake based on *COI* sequences. The colors in the inner ring represent different taxonomic Orders (e.g., Diptera, Odonata, Hemiptera, etc., as shown in the top-right key), while the text labels on the ring denote the corresponding Families (e.g., Chironomidae, Libellulidae).

A total of 104 species were identified, belonging to 74 genera (three genera of Ceratopogonidae remained unidentified), 33 families, and covering eight insect orders (Figure 2). Among them, Diptera was the most species-rich, with 211 sequences obtained, distributed across seven families, with Chironomidae being the most diverse. Odonata ranked second (55 sequences, seven families), followed by Coleoptera (18 sequences, six families) and Hemiptera (13 sequences, six families). In addition, representatives of Ephemeroptera (eight sequences, two families), Trichoptera (six sequences, three families), Lepidoptera (three sequences, one family), and Neuroptera (one sequence, one family) were also detected.

### 3.2. Phylogenetic Tree

The NJ tree provided a general clustering pattern that was consistent with recognized morphology-based groups of aquatic insects, with most major groups recovered as monophyletic (Figure 2). At the order level, Coleoptera, Diptera, Odonata, Ephemeroptera, Hemiptera, Trichoptera, Lepidoptera, and Neuroptera were each distinctly separated and formed independent clades. Among them, Diptera and Odonata were the most species-rich.

Within Diptera, Chironomidae occupied a large and clearly defined monophyletic lineage positioned on the left half of the tree, showing dense terminal branches and extensive taxon representation. Other dipteran families such as Chaoboridae, Ceratopogonidae, and Culicidae were placed on separate branches.

Most families (e.g., Carabidae, Dytiscidae, Ephemeridae, Hydropsychidae) were recovered as discrete lineages with clear branch boundaries. Several groups such as Trichoptera and Lepidoptera were arranged adjacent to one another, whereas Hemiptera and Neuroptera occupied smaller clusters. Overall, the Chironomidae forming one of the most extensive sections, while groups such as Neuroptera and Ephmeroptera comprised fewer taxa.

### 3.3. Genetic Differentiation and Barcode Gap Analysis

**Figure 3:**
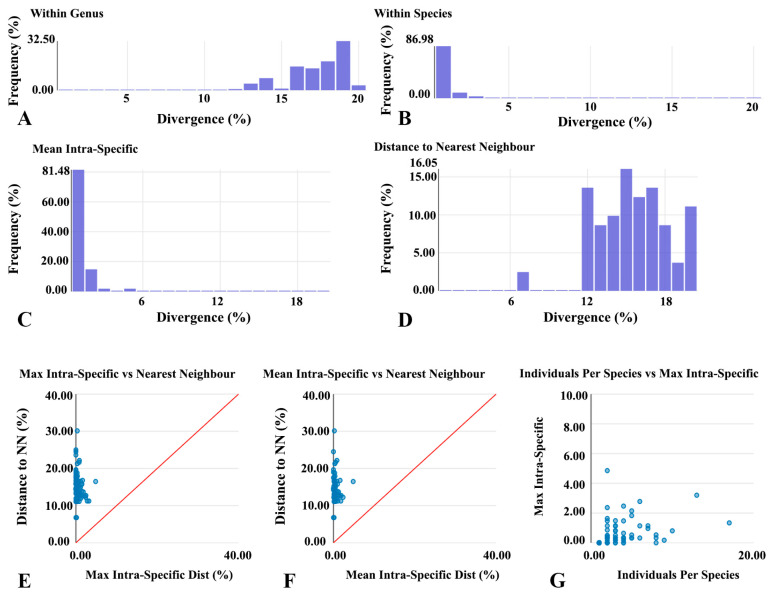
Genetic differentiation and DNA barcode gap analysis of aquatic insects from Baiyangdian Lake. (**A**) Distribution of genetic distances within genera; (**B**) Distribution within species; (**C**) Distribution of per-species mean intraspecific distance; (**D**) Distribution of nearest-neighbor distances; (**E**) Relationship between maximum intraspecific distance and nearest-neighbor distance; (**F**) Relationship between mean intraspecific distance and nearest-neighbor distance; (**G**) Relationship between sample size per species and maximum intraspecific distance.

At the genus level, 883 comparisons produced an average genetic distance of 16.74% (range 6.78–20.85%), mainly concentrated between 15–20% (Figure 3A). At the species level, 622 pairwise comparisons yielded an average intraspecific genetic distance of 0.46% (range 0–4.85%), with 86.98% of comparisons falling below 1% (Figure 3B). Histogram analyses further indicated that over 81% of intraspecific distances were below 1% (Figure 3C,D). Scatter plots demonstrated that the maximum intraspecific distances of all species were lower than their nearest-neighbor distances (Figure 3E). Specifically, the barcode gap analysis identified distinct genetic proximity patterns across the studied orders. In Coleoptera, *Hydroglyphus* and *Ilybius* were identified as nearest neighbors with a substantial genetic distance of 17.39%. Within the dominant family Chironomidae (Diptera), species of *Procladius* frequently showed *Tanypus* (11.16%) and *Sympotthastia* (11.2%) as their closest relatives. Similarly, in Odonata, *Paracercion* species consistently exhibited the closest genetic distances to congenerics (approximately 11.78%), yet these values remained significantly higher than their maximum intraspecific divergences (0.18–0.53%). Consequently, mean intraspecific distances were similarly smaller than nearest-neighbor values (Figure 3F). A positive trend was observed between the maximum intraspecific distance and the number of individuals per species (Figure 3G).

### 3.4. Sampling Sufficiency and Accumulation Curves

**Figure 4:**
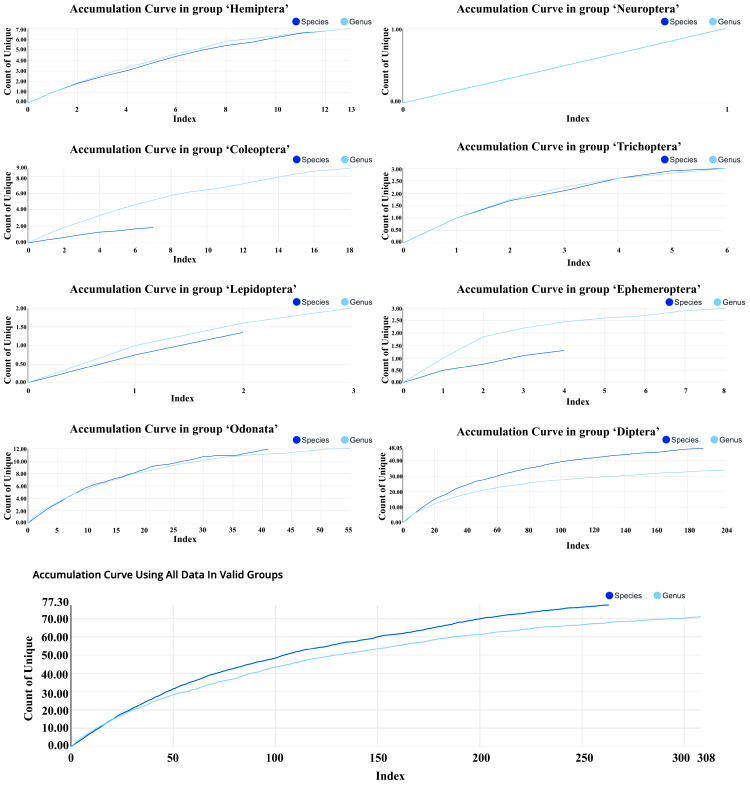
Accumulation curves of aquatic insects from Baiyangdian Lake. Curves show cumulative species- level and genus-level taxa with increasing sequence numbers.

Accumulation curve analysis showed that the total number of unique species-level taxa ultimately reached about 76, while the number of genera reached 70 (Figure 4). With increasing sample size, both species-level and genus-level accumulation curves displayed a “rapid increase and gradual leveling-off” pattern. Early sampling rapidly added taxa, whereas later sampling mainly contributed rare taxa. Notably, the species-level curve continued to rise slightly toward the end and had not fully plateaued; In contrast, the genus-level curve tended to stabilize.

Accumulation curves differed significantly among insect orders. Diptera had the largest sample size (204 sequences), with approximately 48 species-level taxa accumulated; its curve had not yet converged. Odonata accumulated around 12 taxa, with its curve approaching a plateau. Coleoptera (18 sequences) accumulated 2–3 taxa, with genus-level diversity still showing some potential for increase. Hemiptera reached about six taxa, with its curve gradually flattening.

For rare groups, accumulation curves remained low overall. Ephemeroptera and Trichoptera each accumulated 2–3 taxa, with curves rapidly approaching a plateau. Lepidoptera was represented by only 1–2 taxa, and Neuroptera by just one taxon.

## 4. Discussion

### 4.1. Species Diversity and Community Characteristics

The results of this study show that the aquatic insect community in Baiyangdian Lake exhibits high species diversity and a complex community structure. A total of 104 species were identified, belonging to 74 genera, 33 families, and covering eight insect orders. This highlights the ecological diversity of Baiyangdian Lake as a typical shallow lake on the North China Plain.

In terms of community composition, Diptera and Odonata dominate. Diptera ranked first in both species richness and specimen abundance, especially Chironomidae, which demonstrated strong adaptability and ecological ubiquity in lake ecosystems [57,58]. This group not only plays an essential role in energy flow and material cycling but is also widely regarded as an important indicator of freshwater ecosystem health [59]. Odonata, represented mainly by Libellulidae and Coenagrionidae, serve as top predators in the food web, playing a crucial role in regulating aquatic insect population structure [60].

In contrast, although Coleoptera and Hemiptera contain fewer species, they form stable ecological functional units, representing predatory diving beetles and diverse aquatic heteropterans, respectively. Rare taxa such as Ephemeroptera and Trichoptera, though contributing a relatively small proportion to the community, have strong water-quality indicator value [61,62]. Overall, the aquatic insect community of Baiyangdian Lake is characterized by prominent dominant groups, complementary functional groups, and rare groups with indicator value, reflecting the ecosystem’s complexity and stability.

### 4.2. Effectiveness of DNA Barcoding and Phylogenetic Patterns

The NJ tree constructed from *COI* sequences clearly resolved relationships at the order and family levels of aquatic insects. Most major taxa formed well-supported monophyletic groups, confirming the efficiency and reliability of DNA barcoding for species identification [28]. At the order level, Diptera, Odonata, Ephemeroptera, and Hemiptera were accurately distinguished, with tree topology highly consistent with traditional morphological classification. At the family level, Chironomidae, Dytiscidae, and Hydropsychidae were also recovered as independent clades, further demonstrating the applicability of *COI* sequences in community classification analysis [63].

DNA barcoding is particularly valuable when morphological traits are difficult to discern or when larval stages are involved, providing an important complementary approach [64,65]. However, the reliance on a single marker may introduce bias [66].

Although DNA barcoding excels in rapid and large-scale species identification, its resolution can be limited in certain taxa due to uneven evolutionary rates, sequence conservation, or absence of a clear barcode gap [67]. In such cases, morphology remains indispensable for verifying molecular results and detecting cryptic species [68]. Integrating barcoding with multi-gene data and morphological evidence can further strengthen phylogenetic inference. Thus, combining multiple lines of evidence provides more reliable and comprehensive conclusions in biodiversity assessment and phylogenetic reconstruction [69,70].

Overall, the NJ tree robustly captures the major phylogenetic structure of aquatic insects and supports the utility of DNA barcoding for phylogenetic inference and species delimitation in this dataset.

### 4.3. Genetic Differentiation and the Application Value of the Barcode Gap

At the genetic differentiation level, a clear barcode gap was observed: the mean intraspecific distance was only 0.46%, while the mean interspecific distance to the nearest neighbor exceeded 15%. At higher taxonomic levels, the average genetic distances reached 16.74% at the genus level and 19.39% at the family level, reflecting the progressive accumulation of divergence during phylogenetic evolution. These results demonstrate the strong discriminatory power of the *COI* gene [25].

More than 81% of intraspecific comparisons were below 1%, confirming the stability of *COI* as a barcode in aquatic insect communities [71]. A few species with large sample sizes exhibited higher intraspecific variation (>3%), which may be related to insufficient sampling coverage or wide geographic distributions [72,73].

Therefore, DNA barcoding not only provides a reliable tool for species identification but also offers broad applications in detecting cryptic species, evaluating population genetic structure, and analyzing biogeographic patterns [74]. The results underscore the value of DNA barcoding for studying community-level genetic diversity.

### 4.4. Sampling Adequacy and the Degree of Diversity Revealed

Cumulative curve analysis indicated that genus-level diversity was largely captured, as the curve approached a plateau. In contrast, the species-level curve continued to rise, particularly for Diptera, suggesting that undiscovered rare taxa or potential new species may still exist in Baiyangdian Lake.

For rare groups such as Ephemeroptera, Trichoptera, and Neuroptera, the curves leveled off quickly, showing that limited samples could represent their diversity to some extent, though omissions cannot be excluded. A notable case is the mayfly *Choroterpes aprilis*, which was first recorded in Baiyangdian Lake in April 2024 as a new national record for China, confirmed by both morphology and *COI* barcoding [23]. This example highlights that rare groups may harbor unrecognized diversity.

Overall, these results suggest that while the current dataset provides a representative picture of community structure, species-level diversity remains incomplete. Future studies should expand temporal and spatial sampling, particularly in microhabitats such as wetland margins and seasonal water bodies, to uncover additional biodiversity [75].

## 5. Conclusions

This study systematically assessed aquatic insect diversity in Baiyangdian Lake using *COI* DNA barcoding. A total of 104 species from 74 genera and 33 families were identified, with Diptera showing the highest diversity. The NJ tree demonstrated strong concordance between barcodes and morphological taxonomy, while genetic distance and barcode gap analyses confirmed the high discriminatory power of *COI* and indicated the potential existence of cryptic taxa. Accumulation curves suggested that genus-level diversity is largely captured, but species-level diversity, particularly in Diptera, remains incomplete. Overall, this study provides the first comprehensive DNA barcode reference for Baiyangdian aquatic insects, offering a valuable resource for biodiversity monitoring, ecological restoration assessment, and long-term wetland conservation.

## Data Availability

The data presented in this study are openly available in Barcode of Life Data System (http://www.boldsystems.org, 31 October 2025) under the dataset “DNA barcodes of Aquatic insects from Baiyangdian Lake, Hebei, China (DS-BYDAI)”.

## Supplementary Materials

The following supporting information can be downloaded at https://www.mdpi.com/article/10.3390/insects17010060/s1: Table S1: Sample information and collection metadata for aquatic insects in this study.

## Appendix A

**Table A1:** PCR Reaction Mixture.

Protocol	Reagent	Volume
PCR Reaction Mixture	2× Es Taq Master Mix (Dye)	25 µL
	LCO Primer (10 μmol·L^−1^)	2 µL
	HCO Primer (10 μmol·L^−1^)	2 µL
	ddH_2_O	16 µL
	Template DNA	5 µL

**Table A2:** PCR Amplification Program.

Step	Temperature	Purpose	Time	Cycle(s)
Pre-denaturation	94 °C	Initial Denaturation	1 min	1
Cycling 1				5
	94 °C	Denaturation	1 min	
	45 °C	Annealing	90 s	
	72 °C	Extension	90 s	
Cycling 2				35
	94 °C	Denaturation	1 min	
	51 °C	Annealing	90 s	
	72 °C	Extension	1 min	
Final Extension	72 °C	Final Extension	5 min	1
Hold	4 °C	Storage	Forever	1
