# Supplementary material for: Decoding Biodiversity in Baiyangdian Lake: A DNA Barcode Reference Library for Aquatic Insects
Source: Insects. 2026 Jan 1;17(1):60. doi: 10.3390/insects17010060 (PMC12841666; doi:10.3390/insects17010060)
Supplement: Supplementary file 1 [file insects-17-00060-s001.zip › Supplementary File/Table S1.pdf]

Table S1. Sample Information and Collection Metadata for Aquatic Insects in Baiyangdian Lake																	
Sample ID	Phylum	Class	Order	Family	Subfamily	Tribe	Genus	Species	Life Stage	Collectors	Collection Date	Country	Province	Region	Lat	Lon	Elev	Sampling Protocol
BYD650	Arthropoda	Insecta	Coleoptera	Curculionidae	Bagoinae	/	*Bagous*	*Bagous* sp. 1XA	Adult	Yuan Yao	06-Nov-2024	China	Hebei	Xiong`an New Area	38.8228	115.8721	5	D-net
BYD510	Arthropoda	Insecta	Coleoptera	Hydrophilidae	Hydrophilinae	Berosini	*Berosus*	*Berosus lewisius*	Larva	Xiuru Xiao	02-Aug-2024	China	Hebei	Xiong`an New Area	38.9160	115.7430	10	D-net
BYD779	Arthropoda	Insecta	Coleoptera	Chrysomelidae	Donaciinae	/	*Donacia*	*Donacia* sp. 1XA	Larva	Yuan Yao	08-May-2025	China	Hebei	Xiong`an New Area	38.9441	116.0151	9	D-net
BYD925	Arthropoda	Insecta	Coleoptera	Hydrophilidae	Enochrinae	/	*Enochrus*	*Enochrus simulans*	Adult	Yuan Yao	15-May-2025	China	Hebei	Xiong`an New Area	38.9035	115.7929	10	D-net
BYD466	Arthropoda	Insecta	Coleoptera	Hydrophilidae	Enochrinae	/	*Enochrus*	*Enochrus melanocephalus*	Larva	Xiuru Xiao	07-Aug-2024	China	Hebei	Xiong`an New Area	38.9170	115.8200	8	D-net
BYD698	Arthropoda	Insecta	Coleoptera	Dytiscidae	Hydroporinae	Bidessini	*Hydroglyphus*	*Hydroglyphus geminus*	Adult	Yuan Yao	06-Nov-2024	China	Hebei	Xiong`an New Area	38.7650	116.0195	7	D-net
BYD995	Arthropoda	Insecta	Coleoptera	Dytiscidae	Agabinae	Agabini	*Ilybius*	*Ilybius apicalis*	Adult	Yuan Yao	14-May-2025	China	Hebei	Xiong`an New Area	38.7651	116.0194	7	D-net
BYD97	Arthropoda	Insecta	Coleoptera	Dytiscidae	Agabinae	Agabini	*Ilybius*	*Ilybius apicalis*	Adult	Xiaolong Lin	18-Jan-2024	China	Hebei	Xiong`an New Area	38.9190	115.8430	6	D-net
BYD591	Arthropoda	Insecta	Coleoptera	Dytiscidae	Agabinae	Agabini	*Ilybius*	*Ilybius apicalis*	Larva	Yuan Yao	06-Nov-2024	China	Hebei	Xiong`an New Area	38.9498	115.9576	6	D-net
BYD506	Arthropoda	Insecta	Coleoptera	Dytiscidae	Laccophilinae	Laccophilini	*Laccophilus*	*Laccophilus* sp. 1XA	Larva	Xiuru Xiao	04-Aug-2024	China	Hebei	Xiong`an New Area	38.8980	116.0950	7	D-net
BYD296	Arthropoda	Insecta	Coleoptera	Dytiscidae	Laccophilinae	Laccophilini	*Laccophilus*	*Laccophilus* sp. 1XA	Larva	Xiaolong Lin	17-Apr-2024	China	Hebei	Xiong`an New Area	38.8400	115.9510	6	D-net
BYD292	Arthropoda	Insecta	Coleoptera	Dytiscidae	Laccophilinae	Laccophilini	*Laccophilus*	*Laccophilus* sp. 1XA	Larva	Xiaolong Lin	17-Apr-2024	China	Hebei	Xiong`an New Area	38.8490	115.9500	6	D-net
BYD884	Arthropoda	Insecta	Coleoptera	Noteridae	Noterinae	/	*Noterus*	*Noterus* sp. 1XA	Adult	Yuan Yao	12-May-2025	China	Hebei	Xiong`an New Area	38.9148	115.8216	8	D-net
BYD923	Arthropoda	Insecta	Coleoptera	Haliplidae	/	/	*Peltodytes*	*Peltodytes* sp. 1XA	Larva	Yuan Yao	15-May-2025	China	Hebei	Xiong`an New Area	38.9035	115.7929	10	D-net
BYD920	Arthropoda	Insecta	Coleoptera	Haliplidae	/	/	*Peltodytes*	*Peltodytes* sp. 2XA	Larva	Yuan Yao	15-May-2025	China	Hebei	Xiong`an New Area	38.9035	115.7929	10	D-net
BYD487	Arthropoda	Insecta	Coleoptera	Haliplidae	/	/	*Peltodytes*	*Peltodytes* sp. 1XA	Larva	Xiuru Xiao	01-Aug-2024	China	Hebei	Xiong`an New Area	38.8070	115.8440	10	D-net
BYD486	Arthropoda	Insecta	Coleoptera	Haliplidae	/	/	*Peltodytes*	*Peltodytes* sp. 1XA	Larva	Xiuru Xiao	01-Aug-2024	China	Hebei	Xiong`an New Area	38.8070	115.8440	10	D-net
BYD375	Arthropoda	Insecta	Coleoptera	Haliplidae	/	/	*Peltodytes*	*Peltodytes* sp. 1XA	Larva	Xiaolong Lin	14-Apr-2024	China	Hebei	Xiong`an New Area	38.7650	116.0200	6	D-net
BYD988	Arthropoda	Insecta	Diptera	Chironomidae	Tanypodinae	Pentaneurini	*Ablabesmyia*	*Ablabesmyia monilis*	Larva	Yuan Yao	17-May-2025	China	Hebei	Xiong`an New Area	38.8109	115.9853	8	D-net
BYD909	Arthropoda	Insecta	Diptera	Chironomidae	Tanypodinae	Pentaneurini	*Ablabesmyia*	*Ablabesmyia monilis*	Larva	Yuan Yao	15-May-2025	China	Hebei	Xiong`an New Area	38.9035	115.7929	10	D-net
BYD870	Arthropoda	Insecta	Diptera	Chironomidae	Tanypodinae	Pentaneurini	*Ablabesmyia*	*Ablabesmyia monilis*	Larva	Yuan Yao	11-May-2025	China	Hebei	Xiong`an New Area	38.8601	115.9890	8	D-net
BYD812	Arthropoda	Insecta	Diptera	Chironomidae	Tanypodinae	Pentaneurini	*Ablabesmyia*	*Ablabesmyia prorasha*	Larva	Yuan Yao	07-May-2025	China	Hebei	Xiong`an New Area	38.9982	116.0224	8	D-net
BYD771	Arthropoda	Insecta	Diptera	Chironomidae	Tanypodinae	Pentaneurini	*Ablabesmyia*	*Ablabesmyia monilis*	Larva	Yuan Yao	07-May-2025	China	Hebei	Xiong`an New Area	38.9840	115.9762	10	D-net
BYD434	Arthropoda	Insecta	Diptera	Culicidae	Anophelinae	/	*Anopheles*	*Anopheles sinensis*	Larva	Xiuru Xiao	07-Aug-2024	China	Hebei	Xiong`an New Area	38.9240	115.8750	8	D-net
BYD363	Arthropoda	Insecta	Diptera	Ceratopogonidae	Ceratopogoninae	Palpomyiini	*Bezzia*	*Bezzia* sp. 1XA	Larva	Xiaolong Lin	14-Apr-2024	China	Hebei	Xiong`an New Area	38.7770	115.8560	6	D-net
BYD488	Arthropoda	Insecta	Diptera	Stratiomyidae	Stratiomyinae	Oxycerini	*Caloparyphus*	*Caloparyphus* sp. 1XA	Larva	Xiuru Xiao	01-Aug-2024	China	Hebei	Xiong`an New Area	38.8070	115.8440	10	D-net
BYD854	Arthropoda	Insecta	Diptera	Chaoboridae	Chaoborinae	/	*Chaoborus*	*Chaoborus flavicans*	Larva	Yuan Yao	11-May-2025	China	Hebei	Xiong`an New Area	38.8625	116.0307	8	D-net
BYD499	Arthropoda	Insecta	Diptera	Chaoboridae	Chaoborinae	/	*Chaoborus*	*Chaoborus flavicans*	Larva	Xiuru Xiao	05-Aug-2024	China	Hebei	Xiong`an New Area	38.8120	115.9850	6	D-net
BYD498	Arthropoda	Insecta	Diptera	Chaoboridae	Chaoborinae	/	*Chaoborus*	*Chaoborus flavicans*	Larva	Xiuru Xiao	05-Aug-2024	China	Hebei	Xiong`an New Area	38.8120	115.9850	6	D-net
BYD497	Arthropoda	Insecta	Diptera	Chaoboridae	Chaoborinae	/	*Chaoborus*	*Chaoborus flavicans*	Larva	Xiuru Xiao	05-Aug-2024	China	Hebei	Xiong`an New Area	38.8440	115.9900	5	D-net
BYD984	Arthropoda	Insecta	Diptera	Chironomidae	Chironominae	Chironomini	*Chironomus*	*Chironomus dissidens*	Larva	Yuan Yao	17-May-2025	China	Hebei	Xiong`an New Area	38.7898	116.0160	6	D-net
BYD972	Arthropoda	Insecta	Diptera	Chironomidae	Chironominae	Chironomini	*Chironomus*	*Chironomus dissidens*	Larva	Yuan Yao	16-May-2025	China	Hebei	Xiong`an New Area	38.8269	116.0100	5	D-net
BYD950	Arthropoda	Insecta	Diptera	Chironomidae	Chironominae	Chironomini	*Chironomus*	*Chironomus dissidens*	Larva	Yuan Yao	16-May-2025	China	Hebei	Xiong`an New Area	38.8421	116.0242	9	D-net
BYD861	Arthropoda	Insecta	Diptera	Chironomidae	Chironominae	Chironomini	*Chironomus*	*Chironomus tentans*	Larva	Yuan Yao	11-May-2025	China	Hebei	Xiong`an New Area	38.8719	116.0592	5	D-net
BYD847	Arthropoda	Insecta	Diptera	Chironomidae	Chironominae	Chironomini	*Chironomus*	*Chironomus plumosus*	Pupa	Yuan Yao	10-May-2025	China	Hebei	Xiong`an New Area	38.9080	116.0040	8	D-net
BYD824	Arthropoda	Insecta	Diptera	Chironomidae	Chironominae	Chironomini	*Chironomus*	*Chironomus tentans*	Larva	Yuan Yao	10-May-2025	China	Hebei	Xiong`an New Area	38.8956	116.0513	7	D-net
BYD818	Arthropoda	Insecta	Diptera	Chironomidae	Chironominae	Chironomini	*Chironomus*	*Chironomus tentans*	Larva	Yuan Yao	08-May-2025	China	Hebei	Xiong`an New Area	38.9292	116.0278	6	D-net
BYD801	Arthropoda	Insecta	Diptera	Chironomidae	Chironominae	Chironomini	*Chironomus*	*Chironomus dissidens*	Pupa	Yuan Yao	07-May-2025	China	Hebei	Xiong`an New Area	38.9500	115.9570	6	D-net
BYD749	Arthropoda	Insecta	Diptera	Chironomidae	Chironominae	Chironomini	*Chironomus*	*Chironomus dissidens*	Larva	Yuan Yao	08-May-2025	China	Hebei	Xiong`an New Area	38.9188	116.0087	7	D-net
BYD745	Arthropoda	Insecta	Diptera	Chironomidae	Chironominae	Chironomini	*Chironomus*	*Chironomus tentans*	Larva	Yuan Yao	08-May-2025	China	Hebei	Xiong`an New Area	38.9188	116.0087	7	D-net
BYD666	Arthropoda	Insecta	Diptera	Chironomidae	Chironominae	Chironomini	*Chironomus*	*Chironomus dissidens*	Larva	Yuan Yao	06-Nov-2024	China	Hebei	Xiong`an New Area	38.8998	116.0261	5	D-net
BYD620	Arthropoda	Insecta	Diptera	Chironomidae	Chironominae	Chironomini	*Chironomus*	*Chironomus dissidens*	Larva	Yuan Yao	06-Nov-2024	China	Hebei	Xiong`an New Area	38.9241	115.8753	4	D-net
BYD566	Arthropoda	Insecta	Diptera	Chironomidae	Chironominae	Chironomini	*Chironomus*	*Chironomus dissidens*	Larva	Yuan Yao	06-Nov-2024	China	Hebei	Xiong`an New Area	38.9037	115.9460	8	D-net
BYD550	Arthropoda	Insecta	Diptera	Chironomidae	Chironominae	Chironomini	*Chironomus*	*Chironomus tentans*	Larva	Yuan Yao	06-Nov-2024	China	Hebei	Xiong`an New Area	38.9488	115.9605	7	D-net
BYD54	Arthropoda	Insecta	Diptera	Chironomidae	Chironominae	Chironomini	*Chironomus*	*Chironomus plumosus*	Larva	Xiaolong Lin	20-Jan-2024	China	Hebei	Xiong`an New Area	38.9440	116.0150	6	D-net
BYD496	Arthropoda	Insecta	Diptera	Chironomidae	Chironominae	Chironomini	*Chironomus*	*Chironomus tentans*	Larva	Xiuru Xiao	05-Aug-2024	China	Hebei	Xiong`an New Area	38.8440	115.9900	5	D-net
BYD460	Arthropoda	Insecta	Diptera	Chironomidae	Chironominae	Chironomini	*Chironomus*	*Chironomus striatipennis*	Larva	Xiuru Xiao	07-Aug-2024	China	Hebei	Xiong`an New Area	38.9170	115.8200	8	D-net
BYD459	Arthropoda	Insecta	Diptera	Chironomidae	Chironominae	Chironomini	*Chironomus*	*Chironomus striatipennis*	Larva	Xiuru Xiao	07-Aug-2024	China	Hebei	Xiong`an New Area	38.9170	115.8200	8	D-net
BYD425	Arthropoda	Insecta	Diptera	Chironomidae	Chironominae	Chironomini	*Chironomus*	*Chironomus dissidens*	Larva	Xiuru Xiao	31-Jul-2024	China	Hebei	Xiong`an New Area	38.9500	115.9580	6	D-net
BYD422	Arthropoda	Insecta	Diptera	Chironomidae	Chironominae	Chironomini	*Chironomus*	*Chironomus dissidens*	Larva	Xiuru Xiao	31-Jul-2024	China	Hebei	Xiong`an New Area	38.9500	115.9580	6	D-net
BYD413	Arthropoda	Insecta	Diptera	Chironomidae	Chironominae	Chironomini	*Chironomus*	*Chironomus dissidens*	Larva	Xiuru Xiao	31-Jul-2024	China	Hebei	Xiong`an New Area	38.9860	115.9700	10	D-net

BYD25	Arthropoda	Insecta	Diptera	Chironomidae	Chironominae	Chironomini	*Chironomus*	*Chironomus tentans*	Larva	Xiaolong Lin	16-Jan-2024	China	Hebei	Xiong`an New Area	38.8960	116.0450	6	D-net
BYD205	Arthropoda	Insecta	Diptera	Chironomidae	Chironominae	Chironomini	*Chironomus*	*Chironomus dissidens*	Larva	Xiaolong Lin	13-Apr-2024	China	Hebei	Xiong`an New Area	38.9490	115.9610	6	D-net
BYD199	Arthropoda	Insecta	Diptera	Chironomidae	Chironominae	Chironomini	*Chironomus*	*Chironomus tentans*	Larva	Xiaolong Lin	13-Apr-2024	China	Hebei	Xiong`an New Area	38.9490	115.9610	6	D-net
BYD188	Arthropoda	Insecta	Diptera	Chironomidae	Chironominae	Chironomini	*Chironomus*	*Chironomus plumosus*	Larva	Xiaolong Lin	14-Apr-2024	China	Hebei	Xiong`an New Area	38.9630	116.0060	6	D-net
BYD149	Arthropoda	Insecta	Diptera	Chironomidae	Chironominae	Chironomini	*Chironomus*	*Chironomus striatipennis*	Larva	Mengyu Lv	01-Nov-2023	China	Hebei	Xiong`an New Area	38.9250	115.8750	6	D-net
BYD142	Arthropoda	Insecta	Diptera	Chironomidae	Chironominae	Chironomini	*Chironomus*	*Chironomus dissidens*	Larva	Mengyu Lv	01-Nov-2023	China	Hebei	Xiong`an New Area	38.8870	116.0930	6	D-net
BYD121	Arthropoda	Insecta	Diptera	Chironomidae	Chironominae	Chironomini	*Chironomus*	*Chironomus plumosus*	Larva	Xiaolong Lin	20-Jan-2024	China	Hebei	Xiong`an New Area	38.9170	115.8200	6	D-net
BYD1012	Arthropoda	Insecta	Diptera	Chironomidae	Chironominae	Chironomini	*Chironomus*	*Chironomus plumosus*	Larva	Yuan Yao	16-May-2025	China	Hebei	Xiong`an New Area	38.8278	115.9932	5	D-net
BYD1008	Arthropoda	Insecta	Diptera	Chironomidae	Chironominae	Chironomini	*Chironomus*	*Chironomus striatipennis*	Larva	Yuan Yao	16-May-2025	China	Hebei	Xiong`an New Area	38.8040	115.7740	7	D-net
BYD964	Arthropoda	Insecta	Diptera	Chironomidae	Chironominae	Chironomini	*Cladopelma*	*Cladopelma virescens*	Larva	Yuan Yao	14-May-2025	China	Hebei	Xiong`an New Area	38.8084	115.8518	3	D-net
BYD322	Arthropoda	Insecta	Diptera	Chironomidae	Chironominae	Chironomini	*Cladopelma*	*Cladopelma virescens*	Larva	Xiaolong Lin	16-Apr-2024	China	Hebei	Xiong`an New Area	38.8330	116.0210	6	D-net
BYD695	Arthropoda	Insecta	Diptera	Chironomidae	Tanypodinae	Clinotanypodini	*Clinotanypus*	*Clinotanypus yani*	Larva	Yuan Yao	06-Nov-2024	China	Hebei	Xiong`an New Area	38.7765	115.8555	7	D-net
BYD992	Arthropoda	Insecta	Diptera	Chironomidae	Orthocladiinae	/	*Corynoneura*	*Corynoneura* sp. K1	Larva	Yuan Yao	14-May-2025	China	Hebei	Xiong`an New Area	38.7651	116.0194	7	D-net
BYD977	Arthropoda	Insecta	Diptera	Chironomidae	Orthocladiinae	/	*Corynoneura*	*Corynoneura* sp. K1	Larva	Yuan Yao	16-May-2025	China	Hebei	Xiong`an New Area	38.8846	115.8188	10	D-net
BYD957	Arthropoda	Insecta	Diptera	Chironomidae	Orthocladiinae	/	*Corynoneura*	*Corynoneura* sp. K1	Larva	Yuan Yao	14-May-2025	China	Hebei	Xiong`an New Area	38.8084	115.8518	3	D-net
BYD955	Arthropoda	Insecta	Diptera	Chironomidae	Orthocladiinae	/	*Corynoneura*	*Corynoneura* sp. K1	Larva	Yuan Yao	14-May-2025	China	Hebei	Xiong`an New Area	38.8084	115.8518	3	D-net
BYD900	Arthropoda	Insecta	Diptera	Chironomidae	Orthocladiinae	/	*Corynoneura*	*Corynoneura* sp. K1	Larva	Yuan Yao	13-May-2025	China	Hebei	Xiong`an New Area	38.8488	115.9492	9	D-net
BYD981	Arthropoda	Insecta	Diptera	Chironomidae	Orthocladiinae	/	*Cricotopus*	*Cricotopus trifasciatus*	Larva	Yuan Yao	16-May-2025	China	Hebei	Xiong`an New Area	38.8846	115.8188	10	D-net
BYD946	Arthropoda	Insecta	Diptera	Chironomidae	Orthocladiinae	/	*Cricotopus*	*Cricotopus trifasciatus*	Larva	Yuan Yao	14-May-2025	China	Hebei	Xiong`an New Area	38.7739	115.8626	10	D-net
BYD852	Arthropoda	Insecta	Diptera	Chironomidae	Orthocladiinae	/	*Cricotopus*	*Cricotopus trifasciatus*	Larva	Yuan Yao	11-May-2025	China	Hebei	Xiong`an New Area	38.8625	116.0307	8	D-net
BYD820	Arthropoda	Insecta	Diptera	Chironomidae	Orthocladiinae	/	*Cricotopus*	*Cricotopus bicinctus*	Pupa	Yuan Yao	08-May-2025	China	Hebei	Xiong`an New Area	38.9292	116.0278	6	D-net
BYD796	Arthropoda	Insecta	Diptera	Chironomidae	Orthocladiinae	/	*Cricotopus*	*Cricotopus trifasciatus*	Larva	Yuan Yao	08-May-2025	China	Hebei	Xiong`an New Area	38.9420	115.9999	7	D-net
BYD793	Arthropoda	Insecta	Diptera	Chironomidae	Orthocladiinae	/	*Cricotopus*	*Cricotopus trifasciatus*	Larva	Yuan Yao	08-May-2025	China	Hebei	Xiong`an New Area	38.9420	115.9999	7	D-net
BYD782	Arthropoda	Insecta	Diptera	Chironomidae	Orthocladiinae	/	*Cricotopus*	*Cricotopus bicinctus*	Larva	Yuan Yao	08-May-2025	China	Hebei	Xiong`an New Area	38.9441	116.0151	9	D-net
BYD404	Arthropoda	Insecta	Diptera	Chironomidae	Orthocladiinae	/	*Cricotopus*	*Cricotopus trifasciatus*	Larva	Xiuru Xiao	07-Aug-2024	China	Hebei	Xiong`an New Area	38.9040	115.9460	9	D-net
BYD314	Arthropoda	Insecta	Diptera	Chironomidae	Orthocladiinae	/	*Cricotopus*	*Cricotopus trifasciatus*	Larva	Xiaolong Lin	14-Apr-2024	China	Hebei	Xiong`an New Area	38.7750	115.8620	6	D-net
BYD298	Arthropoda	Insecta	Diptera	Chironomidae	Orthocladiinae	/	*Cricotopus*	*Cricotopus trifasciatus*	Larva	Xiaolong Lin	17-Apr-2024	China	Hebei	Xiong`an New Area	38.8400	115.9510	6	D-net
BYD286	Arthropoda	Insecta	Diptera	Chironomidae	Orthocladiinae	/	*Cricotopus*	*Cricotopus trifasciatus*	Larva	Xiaolong Lin	18-Apr-2024	China	Hebei	Xiong`an New Area	38.9200	115.8860	6	D-net
BYD1015	Arthropoda	Insecta	Diptera	Chironomidae	Orthocladiinae	/	*Cricotopus*	*Cricotopus trifasciatus*	Larva	Yuan Yao	17-May-2025	China	Hebei	Xiong`an New Area	38.7986	116.0194	8	D-net
BYD885	Arthropoda	Insecta	Diptera	Chironomidae	Chironominae	Chironomini	*Cryptochironomus*	*Cryptochironomus rostratus*	Larva	Yuan Yao	12-May-2025	China	Hebei	Xiong`an New Area	38.9252	115.8755	8	D-net
BYD810	Arthropoda	Insecta	Diptera	Chironomidae	Chironominae	Chironomini	*Cryptochironomus*	*Cryptochironomus rostratus*	Larva	Yuan Yao	07-May-2025	China	Hebei	Xiong`an New Area	38.9982	116.0224	8	D-net
BYD809	Arthropoda	Insecta	Diptera	Chironomidae	Chironominae	Chironomini	*Cryptochironomus*	*Cryptochironomus rostratus*	Pupa	Yuan Yao	07-May-2025	China	Hebei	Xiong`an New Area	38.9694	116.0024	8	D-net
BYD736	Arthropoda	Insecta	Diptera	Chironomidae	Chironominae	Chironomini	*Cryptochironomus*	*Cryptochironomus rostratus*	Larva	Yuan Yao	07-May-2025	China	Hebei	Xiong`an New Area	38.9719	115.9803	7	D-net
BYD577	Arthropoda	Insecta	Diptera	Chironomidae	Chironominae	Chironomini	*Cryptochironomus*	*Cryptochironomus rostratus*	Larva	Yuan Yao	06-Nov-2024	China	Hebei	Xiong`an New Area	38.9862	115.9704	13	D-net
BYD529	Arthropoda	Insecta	Diptera	Chironomidae	Chironominae	Chironomini	*Cryptochironomus*	*Cryptochironomus rostratus*	Larva	Yuan Yao	06-Nov-2024	China	Hebei	Xiong`an New Area	38.9633	116.0063	6	D-net
BYD393	Arthropoda	Insecta	Diptera	Chironomidae	Chironominae	Chironomini	*Cryptochironomus*	*Cryptochironomus rostratus*	Larva	Xiuru Xiao	01-Aug-2024	China	Hebei	Xiong`an New Area	38.9630	116.0060	6	D-net
BYD328	Arthropoda	Insecta	Diptera	Chironomidae	Chironominae	Chironomini	*Cryptochironomus*	*Cryptochironomus rostratus*	Larva	Xiaolong Lin	15-Apr-2024	China	Hebei	Xiong`an New Area	38.9000	116.0260	6	D-net
BYD14	Arthropoda	Insecta	Diptera	Chironomidae	Chironominae	Chironomini	*Cryptochironomus*	*Cryptochironomus rostratus*	Larva	Xiaolong Lin	16-Jan-2024	China	Hebei	Xiong`an New Area	38.9190	116.0090	6	D-net
BYD464	Arthropoda	Insecta	Diptera	Culicidae	Culicinae	Culicini	*Culex*	*Culex* sp. 1XA	Larva	Xiuru Xiao	07-Aug-2024	China	Hebei	Xiong`an New Area	38.9170	115.8200	8	D-net
BYD902	Arthropoda	Insecta	Diptera	Chironomidae	Chironominae	Chironomini	*Dicrotendipes*	*Dicrotendipes pelochloris*	Larva	Yuan Yao	13-May-2025	China	Hebei	Xiong`an New Area	38.8247	115.8936	5	D-net
BYD823	Arthropoda	Insecta	Diptera	Chironomidae	Chironominae	Chironomini	*Dicrotendipes*	*Dicrotendipes pelochloris*	Larva	Yuan Yao	08-May-2025	China	Hebei	Xiong`an New Area	38.9130	116.0465	6	D-net
BYD786	Arthropoda	Insecta	Diptera	Chironomidae	Chironominae	Chironomini	*Dicrotendipes*	*Dicrotendipes* sp. SK-2011	Larva	Yuan Yao	08-May-2025	China	Hebei	Xiong`an New Area	38.9441	116.0151	9	D-net
BYD748	Arthropoda	Insecta	Diptera	Chironomidae	Chironominae	Chironomini	*Dicrotendipes*	*Dicrotendipes pelochloris*	Larva	Yuan Yao	08-May-2025	China	Hebei	Xiong`an New Area	38.9188	116.0087	7	D-net
BYD70	Arthropoda	Insecta	Diptera	Chironomidae	Chironominae	Chironomini	*Dicrotendipes*	*Dicrotendipes* sp. 2XL	Larva	Xiaolong Lin	16-Jan-2024	China	Hebei	Xiong`an New Area	38.9980	116.0220	6	D-net
BYD685	Arthropoda	Insecta	Diptera	Chironomidae	Chironominae	Chironomini	*Dicrotendipes*	*Dicrotendipes* sp. 2XL	Larva	Yuan Yao	06-Nov-2024	China	Hebei	Xiong`an New Area	38.9158	115.7435	10	D-net
BYD648	Arthropoda	Insecta	Diptera	Chironomidae	Chironominae	Chironomini	*Dicrotendipes*	*Dicrotendipes* sp. SK-2011	Larva	Yuan Yao	06-Nov-2024	China	Hebei	Xiong`an New Area	38.8404	115.9515	5	D-net
BYD441	Arthropoda	Insecta	Diptera	Chironomidae	Chironominae	Chironomini	*Dicrotendipes*	*Dicrotendipes pelochloris*	Larva	Xiuru Xiao	02-Aug-2024	China	Hebei	Xiong`an New Area	38.9030	115.7910	7	D-net
BYD355	Arthropoda	Insecta	Diptera	Chironomidae	Chironominae	Chironomini	*Dicrotendipes*	*Dicrotendipes* sp. 2XL	Larva	Xiaolong Lin	13-Apr-2024	China	Hebei	Xiong`an New Area	38.9160	115.7430	6	D-net
BYD354	Arthropoda	Insecta	Diptera	Chironomidae	Chironominae	Chironomini	*Dicrotendipes*	*Dicrotendipes* sp. 2XL	Larva	Xiaolong Lin	13-Apr-2024	China	Hebei	Xiong`an New Area	38.9160	115.7430	6	D-net
BYD353	Arthropoda	Insecta	Diptera	Chironomidae	Chironominae	Chironomini	*Dicrotendipes*	*Dicrotendipes* sp. 2XL	Larva	Xiaolong Lin	13-Apr-2024	China	Hebei	Xiong`an New Area	38.9160	115.7430	6	D-net
BYD247	Arthropoda	Insecta	Diptera	Chironomidae	Chironominae	Chironomini	*Dicrotendipes*	*Dicrotendipes* sp. 2XL	Larva	Xiaolong Lin	13-Apr-2024	China	Hebei	Xiong`an New Area	38.9860	115.9700	6	D-net
BYD590	Arthropoda	Insecta	Diptera	Chironomidae	Chironominae	Chironomini	*Endochironomus*	*Endochironomus pekanus*	Larva	Yuan Yao	06-Nov-2024	China	Hebei	Xiong`an New Area	38.9498	115.9576	6	D-net
BYD490	Arthropoda	Insecta	Diptera	Chironomidae	Chironominae	Chironomini	*Endochironomus*	*Endochironomus pekanus*	Larva	Xiuru Xiao	01-Aug-2024	China	Hebei	Xiong`an New Area	38.8070	115.8440	10	D-net
BYD989	Arthropoda	Insecta	Diptera	Chironomidae	Chironominae	Chironomini	*Glyptotendipes*	*Glyptotendipes tokunagai*	Larva	Yuan Yao	17-May-2025	China	Hebei	Xiong`an New Area	38.8109	115.9853	8	D-net
BYD948	Arthropoda	Insecta	Diptera	Chironomidae	Chironominae	Chironomini	*Glyptotendipes*	*Glyptotendipes tokunagai*	Larva	Yuan Yao	14-May-2025	China	Hebei	Xiong`an New Area	38.7739	115.8626	10	D-net
BYD658	Arthropoda	Insecta	Diptera	Chironomidae	Chironominae	Chironomini	*Glyptotendipes*	*Glyptotendipes* sp. 1XA	Larva	Yuan Yao	06-Nov-2024	China	Hebei	Xiong`an New Area	38.8069	115.8439	10	D-net

BYD586	Arthropoda	Insecta	Diptera	Chironomidae	Chironominae	Chironomini	*Glyptotendipes*	*Glyptotendipes* sp. 2XA	Larva	Yuan Yao	06-Nov-2024	China	Hebei	Xiong`an New Area	38.9862	115.9704	13	D-net
BYD517	Arthropoda	Insecta	Diptera	Chironomidae	Chironominae	Chironomini	*Glyptotendipes*	*Glyptotendipes* sp. 1XA	Larva	Xiuru Xiao	01-Aug-2024	China	Hebei	Xiong`an New Area	38.7770	115.8560	7	D-net
BYD907	Arthropoda	Insecta	Diptera	Chironomidae	Chironominae	Chironomini	*Glyptotendipes*	*Glyptotendipes pallens*	Larva	Yuan Yao	13-May-2025	China	Hebei	Xiong`an New Area	38.8338	115.9660	5	D-net
BYD904	Arthropoda	Insecta	Diptera	Chironomidae	Chironominae	Chironomini	*Glyptotendipes*	*Glyptotendipes pallens*	Larva	Yuan Yao	13-May-2025	China	Hebei	Xiong`an New Area	38.8248	115.8759	5	D-net
BYD899	Arthropoda	Insecta	Diptera	Chironomidae	Chironominae	Chironomini	*Glyptotendipes*	*Glyptotendipes lobiferus*	Larva	Yuan Yao	13-May-2025	China	Hebei	Xiong`an New Area	38.8488	115.9492	9	D-net
BYD882	Arthropoda	Insecta	Diptera	Chironomidae	Chironominae	Chironomini	*Glyptotendipes*	*Glyptotendipes barbipes*	Larva	Yuan Yao	12-May-2025	China	Hebei	Xiong`an New Area	38.9148	115.8216	8	D-net
BYD855	Arthropoda	Insecta	Diptera	Chironomidae	Chironominae	Chironomini	*Glyptotendipes*	*Glyptotendipes lobiferus*	Larva	Yuan Yao	11-May-2025	China	Hebei	Xiong`an New Area	38.8625	116.0307	8	D-net
BYD804	Arthropoda	Insecta	Diptera	Chironomidae	Chironominae	Chironomini	*Glyptotendipes*	*Glyptotendipes lobiferus*	Larva	Yuan Yao	07-May-2025	China	Hebei	Xiong`an New Area	38.9500	115.9570	6	D-net
BYD645	Arthropoda	Insecta	Diptera	Chironomidae	Chironominae	Chironomini	*Glyptotendipes*	*Glyptotendipes tokunagai*	Larva	Yuan Yao	06-Nov-2024	China	Hebei	Xiong`an New Area	38.9201	115.8861	5	D-net
BYD504	Arthropoda	Insecta	Diptera	Chironomidae	Chironominae	Chironomini	*Glyptotendipes*	*Glyptotendipes barbipes*	Larva	Xiuru Xiao	04-Aug-2024	China	Hebei	Xiong`an New Area	38.8680	116.0680	7	D-net
BYD491	Arthropoda	Insecta	Diptera	Chironomidae	Chironominae	Chironomini	*Glyptotendipes*	*Glyptotendipes lobiferus*	Larva	Xiuru Xiao	06-Aug-2024	China	Hebei	Xiong`an New Area	38.8400	115.9190	5	D-net
BYD433	Arthropoda	Insecta	Diptera	Chironomidae	Chironominae	Chironomini	*Glyptotendipes*	*Glyptotendipes tokunagai*	Larva	Xiuru Xiao	07-Aug-2024	China	Hebei	Xiong`an New Area	38.9240	115.8750	8	D-net
BYD412	Arthropoda	Insecta	Diptera	Chironomidae	Chironominae	Chironomini	*Glyptotendipes*	*Glyptotendipes tokunagai*	Larva	Xiuru Xiao	31-Jul-2024	China	Hebei	Xiong`an New Area	38.9860	115.9700	10	D-net
BYD312	Arthropoda	Insecta	Diptera	Chironomidae	Chironominae	Chironomini	*Glyptotendipes*	*Glyptotendipes pallens*	Larva	Xiaolong Lin	17-Apr-2024	China	Hebei	Xiong`an New Area	38.8400	115.9190	6	D-net
BYD311	Arthropoda	Insecta	Diptera	Chironomidae	Chironominae	Chironomini	*Glyptotendipes*	*Glyptotendipes pallens*	Larva	Xiaolong Lin	17-Apr-2024	China	Hebei	Xiong`an New Area	38.8400	115.9190	6	D-net
BYD310	Arthropoda	Insecta	Diptera	Chironomidae	Chironominae	Chironomini	*Glyptotendipes*	*Glyptotendipes pallens*	Larva	Xiaolong Lin	17-Apr-2024	China	Hebei	Xiong`an New Area	38.8400	115.9190	6	D-net
BYD285	Arthropoda	Insecta	Diptera	Chironomidae	Chironominae	Chironomini	*Glyptotendipes*	*Glyptotendipes pallens*	Larva	Xiaolong Lin	18-Apr-2024	China	Hebei	Xiong`an New Area	38.9200	115.8860	6	D-net
BYD262	Arthropoda	Insecta	Diptera	Chironomidae	Chironominae	Chironomini	*Glyptotendipes*	*Glyptotendipes tokunagai*	Larva	Xiaolong Lin	18-Apr-2024	China	Hebei	Xiong`an New Area	38.9240	115.8750	6	D-net
BYD253	Arthropoda	Insecta	Diptera	Chironomidae	Chironominae	Chironomini	*Glyptotendipes*	*Glyptotendipes barbipes*	Larva	Xiaolong Lin	14-Apr-2024	China	Hebei	Xiong`an New Area	38.9500	115.9580	6	D-net
BYD237	Arthropoda	Insecta	Diptera	Chironomidae	Chironominae	Chironomini	*Glyptotendipes*	*Glyptotendipes tokunagai*	Larva	Xiaolong Lin	13-Apr-2024	China	Hebei	Xiong`an New Area	38.9860	115.9700	6	D-net
BYD133	Arthropoda	Insecta	Diptera	Chironomidae	Chironominae	Chironomini	*Glyptotendipes*	*Glyptotendipes lobiferus*	Larva	Mengyu Lv	01-Nov-2023	China	Hebei	Xiong`an New Area	38.9490	115.9610	6	D-net
BYD79	Arthropoda	Insecta	Diptera	Chironomidae	Orthocladiinae	/	*Hydrobaenus*	*Hydrobaenus dentistylus*	Larva	Xiaolong Lin	18-Jan-2024	China	Hebei	Xiong`an New Area	38.8860	115.9970	6	D-net
BYD630	Arthropoda	Insecta	Diptera	Chironomidae	Orthocladiinae	/	*Hydrobaenus*	*Hydrobaenus kondoi*	Larva	Yuan Yao	06-Nov-2024	China	Hebei	Xiong`an New Area	38.9029	115.7905	7	D-net
BYD132	Arthropoda	Insecta	Diptera	Chironomidae	Orthocladiinae	/	*Hydrobaenus*	*Hydrobaenus dentistylus*	Larva	Mengyu Lv	01-Nov-2023	China	Hebei	Xiong`an New Area	38.9490	115.9610	6	D-net
BYD11	Arthropoda	Insecta	Diptera	Chironomidae	Orthocladiinae	/	*Hydrobaenus*	*Hydrobaenus kondoi*	Larva	Xiaolong Lin	16-Jan-2024	China	Hebei	Xiong`an New Area	38.9190	116.0090	6	D-net
BYD99	Arthropoda	Insecta	Diptera	Chironomidae	Chironominae	Chironomini	*Kiefferulus*	*Kiefferulus tainanus*	Larva	Xiaolong Lin	18-Jan-2024	China	Hebei	Xiong`an New Area	38.9190	115.8430	6	D-net
BYD619	Arthropoda	Insecta	Diptera	Chironomidae	Chironominae	Chironomini	*Kiefferulus*	*Kiefferulus tainanus*	Larva	Yuan Yao	06-Nov-2024	China	Hebei	Xiong`an New Area	38.9241	115.8753	4	D-net
BYD615	Arthropoda	Insecta	Diptera	Chironomidae	Chironominae	Chironomini	*Kiefferulus*	*Kiefferulus tainanus*	Larva	Yuan Yao	06-Nov-2024	China	Hebei	Xiong`an New Area	38.9241	115.8753	4	D-net
BYD431	Arthropoda	Insecta	Diptera	Chironomidae	Chironominae	Chironomini	*Kiefferulus*	*Kiefferulus tainanus*	Larva	Xiuru Xiao	07-Aug-2024	China	Hebei	Xiong`an New Area	38.9240	115.8750	8	D-net
BYD968	Arthropoda	Insecta	Diptera	Chironomidae	Chironominae	Chironomini	*Microchironomus*	*Microchironomus tabarui*	Larva	Yuan Yao	16-May-2025	China	Hebei	Xiong`an New Area	38.8454	115.9915	5	D-net
BYD903	Arthropoda	Insecta	Diptera	Chironomidae	Chironominae	Chironomini	*Microchironomus*	*Microchironomus tener*	Larva	Yuan Yao	13-May-2025	China	Hebei	Xiong`an New Area	38.8247	115.8936	5	D-net
BYD891	Arthropoda	Insecta	Diptera	Chironomidae	Chironominae	Chironomini	*Microchironomus*	*Microchironomus tabarui*	Larva	Yuan Yao	12-May-2025	China	Hebei	Xiong`an New Area	38.9096	115.8646	8	D-net
BYD890	Arthropoda	Insecta	Diptera	Chironomidae	Chironominae	Chironomini	*Microchironomus*	*Microchironomus tabarui*	Larva	Yuan Yao	12-May-2025	China	Hebei	Xiong`an New Area	38.9096	115.8646	8	D-net
BYD889	Arthropoda	Insecta	Diptera	Chironomidae	Chironominae	Chironomini	*Microchironomus*	*Microchironomus tabarui*	Larva	Yuan Yao	12-May-2025	China	Hebei	Xiong`an New Area	38.9096	115.8646	8	D-net
BYD185	Arthropoda	Insecta	Diptera	Chironomidae	Chironominae	Chironomini	*Microchironomus*	*Microchironomus tener*	Larva	Xiaolong Lin	14-Apr-2024	China	Hebei	Xiong`an New Area	38.9630	116.0060	6	D-net
BYD424	Arthropoda	Insecta	Diptera	Ceratopogonidae	Ceratopogoninae	Johannsenomyiini	*Nilobezzia*	*Nilobezzia* sp. 1XA	Larva	Xiuru Xiao	31-Jul-2024	China	Hebei	Xiong`an New Area	38.9500	115.9580	6	D-net
BYD418	Arthropoda	Insecta	Diptera	Ceratopogonidae	Ceratopogoninae	Johannsenomyiini	*Nilobezzia*	*Nilobezzia* sp. 1XA	Larva	Xiuru Xiao	31-Jul-2024	China	Hebei	Xiong`an New Area	38.9860	115.9700	10	D-net
BYD416	Arthropoda	Insecta	Diptera	Ceratopogonidae	Ceratopogoninae	Johannsenomyiini	*Nilobezzia*	*Nilobezzia* sp. 1XA	Larva	Xiuru Xiao	31-Jul-2024	China	Hebei	Xiong`an New Area	38.9860	115.9700	10	D-net
BYD917	Arthropoda	Insecta	Diptera	Stratiomyidae	Stratiomyinae	Stratiomyini	*Odontomyia*	*Odontomyia garatas*	Larva	Yuan Yao	15-May-2025	China	Hebei	Xiong`an New Area	38.9035	115.7929	10	D-net
BYD897	Arthropoda	Insecta	Diptera	Chironomidae	Chironominae	Chironomini	*Parachironomus*	*Parachironomus monochromus*	Larva	Yuan Yao	13-May-2025	China	Hebei	Xiong`an New Area	38.8356	115.9274	5	D-net
BYD781	Arthropoda	Insecta	Diptera	Chironomidae	Chironominae	Chironomini	*Parachironomus*	*Parachironomus monochromus*	Larva	Yuan Yao	08-May-2025	China	Hebei	Xiong`an New Area	38.9441	116.0151	9	D-net
BYD755	Arthropoda	Insecta	Diptera	Chironomidae	Chironominae	Chironomini	*Parachironomus*	*Parachironomus monochromus*	Larva	Yuan Yao	08-May-2025	China	Hebei	Xiong`an New Area	38.9188	116.0087	7	D-net
BYD752	Arthropoda	Insecta	Diptera	Chironomidae	Chironominae	Chironomini	*Parachironomus*	*Parachironomus gracilior*	Larva	Yuan Yao	08-May-2025	China	Hebei	Xiong`an New Area	38.9188	116.0087	7	D-net
BYD469	Arthropoda	Insecta	Diptera	Chironomidae	Chironominae	Chironomini	*Parachironomus*	*Parachironomus monochromus*	Larva	Xiuru Xiao	07-Aug-2024	China	Hebei	Xiong`an New Area	38.9200	115.8860	5	D-net
BYD467	Arthropoda	Insecta	Diptera	Chironomidae	Chironominae	Chironomini	*Parachironomus*	*Parachironomus monochromus*	Larva	Xiuru Xiao	07-Aug-2024	China	Hebei	Xiong`an New Area	38.9200	115.8860	5	D-net
BYD451	Arthropoda	Insecta	Diptera	Chironomidae	Chironominae	Chironomini	*Parachironomus*	*Parachironomus monochromus*	Larva	Xiuru Xiao	07-Aug-2024	China	Hebei	Xiong`an New Area	38.9190	115.8430	4	D-net
BYD450	Arthropoda	Insecta	Diptera	Chironomidae	Chironominae	Chironomini	*Parachironomus*	*Parachironomus monochromus*	Larva	Xiuru Xiao	07-Aug-2024	China	Hebei	Xiong`an New Area	38.9190	115.8430	4	D-net
BYD952	Arthropoda	Insecta	Diptera	Chironomidae	Chironominae	Tanytarsini	*Paratanytarsus*	*Paratanytarsus laetipes*	Larva	Yuan Yao	16-May-2025	China	Hebei	Xiong`an New Area	38.8421	116.0242	9	D-net
BYD765	Arthropoda	Insecta	Diptera	Chironomidae	Chironominae	Tanytarsini	*Paratanytarsus*	*Paratanytarsus* sp. 1XA	Larva	Yuan Yao	07-May-2025	China	Hebei	Xiong`an New Area	38.9840	115.9762	10	D-net
BYD474	Arthropoda	Insecta	Diptera	Chironomidae	Chironominae	Tanytarsini	*Paratanytarsus*	*Paratanytarsus* sp. 1XA	Larva	Xiuru Xiao	07-Aug-2024	China	Hebei	Xiong`an New Area	38.9200	115.8860	5	D-net
BYD473	Arthropoda	Insecta	Diptera	Chironomidae	Chironominae	Tanytarsini	*Paratanytarsus*	*Paratanytarsus* sp. 1XA	Larva	Xiuru Xiao	07-Aug-2024	China	Hebei	Xiong`an New Area	38.9200	115.8860	5	D-net
BYD318	Arthropoda	Insecta	Diptera	Chironomidae	Chironominae	Tanytarsini	*Paratanytarsus*	*Paratanytarsus* sp. 1XA	Larva	Xiaolong Lin	14-Apr-2024	China	Hebei	Xiong`an New Area	38.7750	115.8620	6	D-net
BYD1014	Arthropoda	Insecta	Diptera	Chironomidae	Chironominae	Tanytarsini	*Paratanytarsus*	*Paratanytarsus laetipes*	Larva	Yuan Yao	16-May-2025	China	Hebei	Xiong`an New Area	38.8278	115.9932	5	D-net
BYD965	Arthropoda	Insecta	Diptera	Chironomidae	Chironominae	Chironomini	*Polypedilum*	*Polypedilum sordens*	Larva	Yuan Yao	14-May-2025	China	Hebei	Xiong`an New Area	38.8084	115.8518	3	D-net
BYD910	Arthropoda	Insecta	Diptera	Chironomidae	Chironominae	Chironomini	*Polypedilum*	*Polypedilum yongsanensis*	Larva	Yuan Yao	15-May-2025	China	Hebei	Xiong`an New Area	38.9035	115.7929	10	D-net
BYD905	Arthropoda	Insecta	Diptera	Chironomidae	Chironominae	Chironomini	*Polypedilum*	*Polypedilum yongsanensis*	Larva	Yuan Yao	13-May-2025	China	Hebei	Xiong`an New Area	38.8248	115.8759	5	D-net

BYD860	Arthropoda	Insecta	Diptera	Chironomidae	Chironominae	Chironomini	*Polypedilum*	*Polypedilum yongsanensis*	Pupa	Yuan Yao	11-May-2025	China	Hebei	Xiong`an New Area	38.8719	116.0592	5	D-net
BYD856	Arthropoda	Insecta	Diptera	Chironomidae	Chironominae	Chironomini	*Polypedilum*	*Polypedilum yongsanensis*	Larva	Yuan Yao	11-May-2025	China	Hebei	Xiong`an New Area	38.8625	116.0307	8	D-net
BYD830	Arthropoda	Insecta	Diptera	Chironomidae	Chironominae	Chironomini	*Polypedilum*	*Polypedilum sordens*	Larva	Yuan Yao	10-May-2025	China	Hebei	Xiong`an New Area	38.8956	116.0513	7	D-net
BYD806	Arthropoda	Insecta	Diptera	Chironomidae	Chironominae	Chironomini	*Polypedilum*	*Polypedilum yongsanensis*	Larva	Yuan Yao	07-May-2025	China	Hebei	Xiong`an New Area	38.9694	116.0024	8	D-net
BYD774	Arthropoda	Insecta	Diptera	Chironomidae	Chironominae	Chironomini	*Polypedilum*	*Polypedilum yongsanensis*	Larva	Yuan Yao	08-May-2025	China	Hebei	Xiong`an New Area	38.9441	116.0151	9	D-net
BYD773	Arthropoda	Insecta	Diptera	Chironomidae	Chironominae	Chironomini	*Polypedilum*	*Polypedilum yongsanensis*	Larva	Yuan Yao	08-May-2025	China	Hebei	Xiong`an New Area	38.9441	116.0151	9	D-net
BYD768	Arthropoda	Insecta	Diptera	Chironomidae	Chironominae	Chironomini	*Polypedilum*	*Polypedilum cultellatum*	Larva	Yuan Yao	07-May-2025	China	Hebei	Xiong`an New Area	38.9840	115.9762	10	D-net
BYD763	Arthropoda	Insecta	Diptera	Chironomidae	Chironominae	Chironomini	*Polypedilum*	*Polypedilum cultellatum*	Pupa	Yuan Yao	07-May-2025	China	Hebei	Xiong`an New Area	38.9840	115.9762	10	D-net
BYD731	Arthropoda	Insecta	Diptera	Chironomidae	Chironominae	Chironomini	*Polypedilum*	*Polypedilum yongsanensis*	Larva	Yuan Yao	07-May-2025	China	Hebei	Xiong`an New Area	38.9719	115.9803	7	D-net
BYD727	Arthropoda	Insecta	Diptera	Chironomidae	Chironominae	Chironomini	*Polypedilum*	*Polypedilum yongsanensis*	Larva	Yuan Yao	07-May-2025	China	Hebei	Xiong`an New Area	38.9719	115.9803	7	D-net
BYD715	Arthropoda	Insecta	Diptera	Chironomidae	Chironominae	Chironomini	*Polypedilum*	*Polypedilum yongsanensis*	Larva	Yuan Yao	06-May-2025	China	Hebei	Xiong`an New Area	38.9037	115.9458	7	D-net
BYD690	Arthropoda	Insecta	Diptera	Chironomidae	Chironominae	Chironomini	*Polypedilum*	*Polypedilum masudai*	Larva	Yuan Yao	06-Nov-2024	China	Hebei	Xiong`an New Area	38.8845	115.8188	10	D-net
BYD599	Arthropoda	Insecta	Diptera	Chironomidae	Chironominae	Chironomini	*Polypedilum*	*Polypedilum cultellatum*	Larva	Yuan Yao	06-Nov-2024	China	Hebei	Xiong`an New Area	38.9498	115.9576	6	D-net
BYD598	Arthropoda	Insecta	Diptera	Chironomidae	Chironominae	Chironomini	*Polypedilum*	*Polypedilum cultellatum*	Larva	Yuan Yao	06-Nov-2024	China	Hebei	Xiong`an New Area	38.9498	115.9576	6	D-net
BYD538	Arthropoda	Insecta	Diptera	Chironomidae	Chironominae	Chironomini	*Polypedilum*	*Polypedilum yongsanensis*	Larva	Yuan Yao	06-Nov-2024	China	Hebei	Xiong`an New Area	38.9633	116.0063	6	D-net
BYD537	Arthropoda	Insecta	Diptera	Chironomidae	Chironominae	Chironomini	*Polypedilum*	*Polypedilum yongsanensis*	Larva	Yuan Yao	06-Nov-2024	China	Hebei	Xiong`an New Area	38.9633	116.0063	6	D-net
BYD526	Arthropoda	Insecta	Diptera	Chironomidae	Chironominae	Chironomini	*Polypedilum*	*Polypedilum decematoguttatum*	Larva	Yuan Yao	06-Nov-2024	China	Hebei	Xiong`an New Area	38.9633	116.0063	6	D-net
BYD503	Arthropoda	Insecta	Diptera	Chironomidae	Chironominae	Chironomini	*Polypedilum*	*Polypedilum masudai*	Larva	Xiuru Xiao	03-Aug-2024	China	Hebei	Xiong`an New Area	38.9190	116.0090	7	D-net
BYD475	Arthropoda	Insecta	Diptera	Chironomidae	Chironominae	Chironomini	*Polypedilum*	*Polypedilum sordens*	Larva	Xiuru Xiao	07-Aug-2024	China	Hebei	Xiong`an New Area	38.9200	115.8860	5	D-net
BYD439	Arthropoda	Insecta	Diptera	Chironomidae	Chironominae	Chironomini	*Polypedilum*	*Polypedilum yongsanensis*	Larva	Xiuru Xiao	02-Aug-2024	China	Hebei	Xiong`an New Area	38.9030	115.7910	7	D-net
BYD438	Arthropoda	Insecta	Diptera	Chironomidae	Chironominae	Chironomini	*Polypedilum*	*Polypedilum yongsanensis*	Larva	Xiuru Xiao	02-Aug-2024	China	Hebei	Xiong`an New Area	38.9030	115.7910	7	D-net
BYD437	Arthropoda	Insecta	Diptera	Chironomidae	Chironominae	Chironomini	*Polypedilum*	*Polypedilum yongsanensis*	Larva	Xiuru Xiao	02-Aug-2024	China	Hebei	Xiong`an New Area	38.9030	115.7910	7	D-net
BYD402	Arthropoda	Insecta	Diptera	Chironomidae	Chironominae	Chironomini	*Polypedilum*	*Polypedilum sordens*	Larva	Xiuru Xiao	31-Jul-2024	China	Hebei	Xiong`an New Area	38.9490	115.9610	7	D-net
BYD389	Arthropoda	Insecta	Diptera	Chironomidae	Chironominae	Chironomini	*Polypedilum*	*Polypedilum masudai*	Larva	Xiuru Xiao	01-Aug-2024	China	Hebei	Xiong`an New Area	38.9630	116.0060	6	D-net
BYD246	Arthropoda	Insecta	Diptera	Chironomidae	Chironominae	Chironomini	*Polypedilum*	*Polypedilum sordens*	Larva	Xiaolong Lin	13-Apr-2024	China	Hebei	Xiong`an New Area	38.9860	115.9700	6	D-net
BYD238	Arthropoda	Insecta	Diptera	Chironomidae	Chironominae	Chironomini	*Polypedilum*	*Polypedilum yongsanensis*	Larva	Xiaolong Lin	13-Apr-2024	China	Hebei	Xiong`an New Area	38.9860	115.9700	6	D-net
BYD216	Arthropoda	Insecta	Diptera	Chironomidae	Chironominae	Chironomini	*Polypedilum*	*Polypedilum masudai*	Larva	Xiaolong Lin	18-Apr-2024	China	Hebei	Xiong`an New Area	38.9040	115.9460	6	D-net
BYD1003	Arthropoda	Insecta	Diptera	Chironomidae	Chironominae	Chironomini	*Polypedilum*	*Polypedilum yongsanensis*	Larva	Yuan Yao	16-May-2025	China	Hebei	Xiong`an New Area	38.9147	115.7663	10	D-net
BYD671	Arthropoda	Insecta	Diptera	Chironomidae	Tanypodinae	Procladiini	*Procladius*	*Procladius crassinervis*	Larva	Yuan Yao	06-Nov-2024	China	Hebei	Xiong`an New Area	38.8637	116.0317	7	D-net
BYD72	Arthropoda	Insecta	Diptera	Chironomidae	Prodiamesinae	/	*Propsilocerus*	*Propsilocerus akamusi*	Larva	Xiaolong Lin	16-Jan-2024	China	Hebei	Xiong`an New Area	38.9980	116.0220	6	D-net
BYD674	Arthropoda	Insecta	Diptera	Chironomidae	Prodiamesinae	/	*Propsilocerus*	*Propsilocerus paradoxus*	Larva	Yuan Yao	06-Nov-2024	China	Hebei	Xiong`an New Area	38.8984	116.0952	7	D-net
BYD5	Arthropoda	Insecta	Diptera	Chironomidae	Prodiamesinae	/	*Propsilocerus*	*Propsilocerus akamusi*	Larva	Xiaolong Lin	16-Jan-2024	China	Hebei	Xiong`an New Area	38.9190	116.0090	6	D-net
BYD32	Arthropoda	Insecta	Diptera	Chironomidae	Prodiamesinae	/	*Propsilocerus*	*Propsilocerus akamusi*	Larva	Xiaolong Lin	16-Jan-2024	China	Hebei	Xiong`an New Area	38.9860	115.9700	6	D-net
BYD2	Arthropoda	Insecta	Diptera	Chironomidae	Prodiamesinae	/	*Propsilocerus*	*Propsilocerus akamusi*	Larva	Xiaolong Lin	16-Jan-2024	China	Hebei	Xiong`an New Area	38.9190	116.0090	6	D-net
BYD134	Arthropoda	Insecta	Diptera	Chironomidae	Prodiamesinae	/	*Propsilocerus*	*Propsilocerus akamusi*	Larva	Mengyu Lv	01-Nov-2023	China	Hebei	Xiong`an New Area	38.9820	115.9720	6	D-net
BYD868	Arthropoda	Insecta	Diptera	Chironomidae	Orthocladiinae	/	*Psectrocladius*	*Psectrocladius aquatronus*	Larva	Yuan Yao	11-May-2025	China	Hebei	Xiong`an New Area	38.8719	116.0592	5	D-net
BYD866	Arthropoda	Insecta	Diptera	Chironomidae	Orthocladiinae	/	*Psectrocladius*	*Psectrocladius aquatronus*	Larva	Yuan Yao	11-May-2025	China	Hebei	Xiong`an New Area	38.8719	116.0592	5	D-net
BYD865	Arthropoda	Insecta	Diptera	Chironomidae	Orthocladiinae	/	*Psectrocladius*	*Psectrocladius aquatronus*	Larva	Yuan Yao	11-May-2025	China	Hebei	Xiong`an New Area	38.8719	116.0592	5	D-net
BYD179	Arthropoda	Insecta	Diptera	Simuliidae	Simuliinae	Simuliini	*Simulium*	*Simulium erythrocephalum*	Larva	Mengyu Lv	01-Nov-2023	China	Hebei	Xiong`an New Area	38.9160	115.7430	6	D-net
BYD178	Arthropoda	Insecta	Diptera	Simuliidae	Simuliinae	Simuliini	*Simulium*	*Simulium erythrocephalum*	Larva	Mengyu Lv	01-Nov-2023	China	Hebei	Xiong`an New Area	38.9160	115.7430	6	D-net
BYD872	Arthropoda	Insecta	Diptera	Chironomidae	Chironominae	Chironomini	*Stenochironomus*	*Stenochironomus koreanus*	Larva	Yuan Yao	11-May-2025	China	Hebei	Xiong`an New Area	38.8601	115.9890	8	D-net
BYD843	Arthropoda	Insecta	Diptera	Chironomidae	Chironominae	Chironomini	*Stenochironomus*	*Stenochironomus koreanus*	Larva	Yuan Yao	10-May-2025	China	Hebei	Xiong`an New Area	38.8972	116.0896	8	D-net
BYD842	Arthropoda	Insecta	Diptera	Chironomidae	Chironominae	Chironomini	*Stenochironomus*	*Stenochironomus koreanus*	Larva	Yuan Yao	10-May-2025	China	Hebei	Xiong`an New Area	38.8972	116.0896	8	D-net
BYD1010	Arthropoda	Insecta	Diptera	Stratiomyidae	Stratiomyinae	Stratiomyini	*Stratiomys*	*Stratiomys laetimaculata*	Larva	Yuan Yao	16-May-2025	China	Hebei	Xiong`an New Area	38.8040	115.7740	7	D-net
BYD66	Arthropoda	Insecta	Diptera	Chironomidae	Diamesinae	Diamesini	*Sympotthastia*	*Sympotthastia takatensis*	Larva	Xiaolong Lin	16-Jan-2024	China	Hebei	Xiong`an New Area	38.9980	116.0220	6	D-net
BYD41	Arthropoda	Insecta	Diptera	Chironomidae	Diamesinae	Diamesini	*Sympotthastia*	*Sympotthastia takatensis*	Larva	Xiaolong Lin	16-Jan-2024	China	Hebei	Xiong`an New Area	38.9630	116.0060	6	D-net
BYD120	Arthropoda	Insecta	Diptera	Chironomidae	Diamesinae	Diamesini	*Sympotthastia*	*Sympotthastia takatensis*	Larva	Xiaolong Lin	20-Jan-2024	China	Hebei	Xiong`an New Area	38.8230	115.8720	6	D-net
BYD849	Arthropoda	Insecta	Diptera	Chironomidae	Tanypodinae	Tanypodini	*Tanypus*	*Tanypus chinensis*	Larva	Yuan Yao	10-May-2025	China	Hebei	Xiong`an New Area	38.9080	116.0040	8	D-net
BYD415	Arthropoda	Insecta	Diptera	Chironomidae	Tanypodinae	Tanypodini	*Tanypus*	*Tanypus chinensis*	Larva	Xiuru Xiao	31-Jul-2024	China	Hebei	Xiong`an New Area	38.9860	115.9700	10	D-net
BYD340	Arthropoda	Insecta	Diptera	Chironomidae	Tanypodinae	Tanypodini	*Tanypus*	*Tanypus chinensis*	Larva	Xiaolong Lin	16-Apr-2024	China	Hebei	Xiong`an New Area	38.8860	115.9970	6	D-net
BYD30	Arthropoda	Insecta	Diptera	Chironomidae	Tanypodinae	Tanypodini	*Tanypus*	*Tanypus chinensis*	Larva	Xiaolong Lin	16-Jan-2024	China	Hebei	Xiong`an New Area	38.9860	115.9700	6	D-net
BYD146	Arthropoda	Insecta	Diptera	Chironomidae	Tanypodinae	Tanypodini	*Tanypus*	*Tanypus chinensis*	Larva	Mengyu Lv	01-Nov-2023	China	Hebei	Xiong`an New Area	38.8100	116.0340	6	D-net
BYD128	Arthropoda	Insecta	Diptera	Chironomidae	Tanypodinae	Tanypodini	*Tanypus*	*Tanypus chinensis*	Larva	Mengyu Lv	01-Nov-2023	China	Hebei	Xiong`an New Area	38.9210	115.9750	6	D-net
BYD886	Arthropoda	Insecta	Diptera	Chironomidae	Chironominae	Tanytarsini	*Tanytarsus*	*Tanytarsus unagiseptimus*	Larva	Yuan Yao	12-May-2025	China	Hebei	Xiong`an New Area	38.9252	115.8755	8	D-net
BYD848	Arthropoda	Insecta	Diptera	Chironomidae	Chironominae	Tanytarsini	*Tanytarsus*	*Tanytarsus* sp. 1XA	Larva	Yuan Yao	10-May-2025	China	Hebei	Xiong`an New Area	38.9080	116.0040	8	D-net
BYD202	Arthropoda	Insecta	Diptera	Chironomidae	Chironominae	Tanytarsini	*Tanytarsus*	*Tanytarsus unagiseptimus*	Larva	Xiaolong Lin	13-Apr-2024	China	Hebei	Xiong`an New Area	38.9490	115.9610	6	D-net

BYD43	Arthropoda	Insecta	Diptera	Chironomidae	Tanypodinae	Pentaneurini	*Thienemannimyia*	*Thienemannimyia* sp. 3XL	Larva	Xiaolong Lin	16-Jan-2024	China	Hebei	Xiong`an New Area	38.9630	116.0060	6	D-net
BYD347	Arthropoda	Insecta	Diptera	Chironomidae	Tanypodinae	Pentaneurini	*Thienemannimyia*	*Thienemannimyia tripunctata*	Larva	Xiaolong Lin	13-Apr-2024	China	Hebei	Xiong`an New Area	38.9160	115.7430	6	D-net
BYD921	Arthropoda	Insecta	Diptera	Tipulidae	Tipulinae	/	*Tipula*	*Tipula* sp. 1XA	Larva	Yuan Yao	15-May-2025	China	Hebei	Xiong`an New Area	38.9035	115.7929	10	D-net
BYD423	Arthropoda	Insecta	Diptera	Ceratopogonidae	Ceratopogoninae	/	/	*Ceratopogoninae* sp. 3XA	Larva	Xiuru Xiao	31-Jul-2024	China	Hebei	Xiong`an New Area	38.9500	115.9580	6	D-net
BYD148	Arthropoda	Insecta	Diptera	Ceratopogonidae	Ceratopogoninae	/	/	*Ceratopogoninae* sp. 3XA	Larva	Mengyu Lv	01-Nov-2023	China	Hebei	Xiong`an New Area	38.8250	115.8760	6	D-net
BYD833	Arthropoda	Insecta	Diptera	Ceratopogonidae	/	/	/	*Ceratopogonidae* sp. 1XA	Larva	Yuan Yao	10-May-2025	China	Hebei	Xiong`an New Area	38.8956	116.0513	7	D-net
BYD811	Arthropoda	Insecta	Diptera	Ceratopogonidae	/	/	/	*Ceratopogonidae* sp. 2XA	Larva	Yuan Yao	07-May-2025	China	Hebei	Xiong`an New Area	38.9982	116.0224	8	D-net
BYD463	Arthropoda	Insecta	Diptera	Ceratopogonidae	/	/	/	*Ceratopogonidae* sp. 1XA	Larva	Xiuru Xiao	07-Aug-2024	China	Hebei	Xiong`an New Area	38.9170	115.8200	8	D-net
BYD462	Arthropoda	Insecta	Diptera	Ceratopogonidae	/	/	/	*Ceratopogonidae* sp. 1XA	Larva	Xiuru Xiao	07-Aug-2024	China	Hebei	Xiong`an New Area	38.9170	115.8200	8	D-net
BYD461	Arthropoda	Insecta	Diptera	Ceratopogonidae	/	/	/	*Ceratopogonidae* sp. 1XA	Larva	Xiuru Xiao	07-Aug-2024	China	Hebei	Xiong`an New Area	38.9170	115.8200	8	D-net
BYD350	Arthropoda	Insecta	Ephemeroptera	Baetidae	Baetinae	/	*Baetis*	*Baetis majus*	Larva	Xiaolong Lin	13-Apr-2024	China	Hebei	Xiong`an New Area	38.9160	115.7430	6	D-net
BYD712	Arthropoda	Insecta	Ephemeroptera	Caenidae	Caeninae	/	*Caenis*	*Caenis* sp. 1XA	Larva	Yuan Yao	06-May-2025	China	Hebei	Xiong`an New Area	38.9037	115.9458	7	D-net
BYD407	Arthropoda	Insecta	Ephemeroptera	Caenidae	Caeninae	/	*Caenis*	*Caenis* sp. 2XA	Larva	Xiuru Xiao	07-Aug-2024	China	Hebei	Xiong`an New Area	38.9040	115.9460	9	D-net
BYD406	Arthropoda	Insecta	Ephemeroptera	Caenidae	Caeninae	/	*Caenis*	*Caenis* sp. 2XA	Larva	Xiuru Xiao	07-Aug-2024	China	Hebei	Xiong`an New Area	38.9040	115.9460	9	D-net
BYD131	Arthropoda	Insecta	Ephemeroptera	Caenidae	Caeninae	/	*Caenis*	*Caenis* sp. 2XA	Larva	Mengyu Lv	01-Nov-2023	China	Hebei	Xiong`an New Area	38.9490	115.9610	6	D-net
BYD435	Arthropoda	Insecta	Ephemeroptera	Baetidae	Baetinae	/	*Cloeon*	*Cloeon viridulum*	Larva	Xiuru Xiao	07-Aug-2024	China	Hebei	Xiong`an New Area	38.9240	115.8750	8	D-net
BYD295	Arthropoda	Insecta	Ephemeroptera	Baetidae	Baetinae	/	*Cloeon*	*Cloeon viridulum*	Larva	Xiaolong Lin	17-Apr-2024	China	Hebei	Xiong`an New Area	38.8400	115.9510	6	D-net
BYD108	Arthropoda	Insecta	Ephemeroptera	Baetidae	Baetinae	/	*Cloeon*	*Cloeon viridulum*	Larva	Xiaolong Lin	19-Jan-2024	China	Hebei	Xiong`an New Area	38.9030	115.7910	6	D-net
BYD515	Arthropoda	Insecta	Hemiptera	Belostomatidae	Belostomatinae	Diplonychini	*Appasus*	*Appasus japonicus*	Larva	Xiuru Xiao	31-Jul-2024	China	Hebei	Xiong`an New Area	38.8850	115.8190	10	D-net
BYD514	Arthropoda	Insecta	Hemiptera	Belostomatidae	Belostomatinae	Diplonychini	*Appasus*	*Appasus japonicus*	Larva	Xiuru Xiao	02-Aug-2024	China	Hebei	Xiong`an New Area	38.9160	115.7430	10	D-net
BYD448	Arthropoda	Insecta	Hemiptera	Belostomatidae	Belostomatinae	Diplonychini	*Appasus*	*Appasus japonicus*	Larva	Xiuru Xiao	02-Aug-2024	China	Hebei	Xiong`an New Area	38.9030	115.7910	7	D-net
BYD702	Arthropoda	Insecta	Hemiptera	Corixidae	Cymatiainae	/	*Cymatia*	*Cymatia apparens*	Adult	Yuan Yao	06-Nov-2024	China	Hebei	Xiong`an New Area	38.9029	115.7905	7	D-net
BYD623	Arthropoda	Insecta	Hemiptera	Corixidae	Cymatiainae	/	*Cymatia*	*Cymatia apparens*	Adult	Yuan Yao	06-Nov-2024	China	Hebei	Xiong`an New Area	38.9029	115.7905	7	D-net
BYD458	Arthropoda	Insecta	Hemiptera	Naucoridae	Ilyocorinae	/	*Ilyocoris*	*Ilyocoris cimicoides*	Larva	Xiuru Xiao	07-Aug-2024	China	Hebei	Xiong`an New Area	38.9170	115.8200	8	D-net
BYD518	Arthropoda	Insecta	Hemiptera	Nepidae	Nepinae	Nepini	*Laccotrephes*	*Laccotrephes japonensis*	Larva	Xiuru Xiao	01-Aug-2024	China	Hebei	Xiong`an New Area	38.7770	115.8560	7	D-net
BYD180	Arthropoda	Insecta	Hemiptera	Micronectidae	Micronectinae	/	*Micronecta*	*Micronecta* sp. 1XA	Larva	Mengyu Lv	01-Nov-2023	China	Hebei	Xiong`an New Area	38.7790	115.8560	6	D-net
BYD197	Arthropoda	Insecta	Hemiptera	Micronectidae	Micronectinae	/	*Micronecta*	*Micronecta sahlbergii*	Larva	Xiaolong Lin	14-Apr-2024	China	Hebei	Xiong`an New Area	38.9630	116.0060	6	D-net
BYD453	Arthropoda	Insecta	Hemiptera	Pleidae	/	/	*Paraplea*	*Paraplea indistinguenda*	Larva	Xiuru Xiao	07-Aug-2024	China	Hebei	Xiong`an New Area	38.9190	115.8430	4	D-net
BYD452	Arthropoda	Insecta	Hemiptera	Pleidae	/	/	*Paraplea*	*Paraplea indistinguenda*	Larva	Xiuru Xiao	07-Aug-2024	China	Hebei	Xiong`an New Area	38.9190	115.8430	4	D-net
BYD443	Arthropoda	Insecta	Hemiptera	Nepidae	Ranatrinae	Ranatrini	*Ranatra*	*Ranatra unicolor*	Larva	Xiuru Xiao	02-Aug-2024	China	Hebei	Xiong`an New Area	38.9030	115.7910	7	D-net
BYD326	Arthropoda	Insecta	Hemiptera	Nepidae	Ranatrinae	Ranatrini	*Ranatra*	*Ranatra unicolor*	Larva	Xiaolong Lin	16-Apr-2024	China	Hebei	Xiong`an New Area	38.8120	115.9850	6	D-net
BYD281	Arthropoda	Insecta	Lepidoptera	Crambidae	Acentropinae	Nymphulini	*Elophila*	*Elophila turbata*	Larva	Xiaolong Lin	18-Apr-2024	China	Hebei	Xiong`an New Area	38.9190	115.8430	6	D-net
BYD374	Arthropoda	Insecta	Lepidoptera	Crambidae	Acentropinae	/	*Parapoynx*	*Parapoynx* sp. 1XA	Larva	Xiaolong Lin	14-Apr-2024	China	Hebei	Xiong`an New Area	38.7650	116.0200	6	D-net
BYD582	Arthropoda	Insecta	Lepidoptera	Crambidae	Acentropinae	/	*Parapoynx*	*Parapoynx diminutalis*	Larva	Yuan Yao	06-Nov-2024	China	Hebei	Xiong`an New Area	38.9862	115.9704	13	D-net
BYD331	Arthropoda	Insecta	Neuroptera	Sisyridae	Sisyrinae	/	*Sisyra*	*Sisyra nikkoana*	Larva	Xiaolong Lin	16-Apr-2024	China	Hebei	Xiong`an New Area	38.8640	116.0320	6	D-net
BYD494	Arthropoda	Insecta	Odonata	Aeshnidae	Aeshninae	/	*Anax*	*Anax parthenope*	Larva	Xiuru Xiao	05-Aug-2024	China	Hebei	Xiong`an New Area	38.8270	116.0200	6	D-net
BYD379	Arthropoda	Insecta	Odonata	Aeshnidae	Aeshninae	/	*Anax*	*Anax parthenope*	Larva	Xiaolong Lin	16-Apr-2024	China	Hebei	Xiong`an New Area	38.8270	116.0200	6	D-net
BYD171	Arthropoda	Insecta	Odonata	Aeshnidae	Aeshninae	/	*Anax*	*Anax parthenope*	Larva	Mengyu Lv	01-Nov-2023	China	Hebei	Xiong`an New Area	38.7480	116.0350	6	D-net
BYD177	Arthropoda	Insecta	Odonata	Calopterygidae	Calopteryginae	/	*Atrocalopteryx*	*Atrocalopteryx atrata*	Larva	Mengyu Lv	01-Nov-2023	China	Hebei	Xiong`an New Area	38.9160	115.7430	6	D-net
BYD1006	Arthropoda	Insecta	Odonata	Calopterygidae	Calopteryginae	/	*Atrocalopteryx*	*Atrocalopteryx atrata*	Larva	Yuan Yao	16-May-2025	China	Hebei	Xiong`an New Area	38.9147	115.7663	10	D-net
BYD836	Arthropoda	Insecta	Odonata	Libellulidae	Sympetrinae	/	*Crocothemis*	*Crocothemis servilia*	Larva	Yuan Yao	10-May-2025	China	Hebei	Xiong`an New Area	38.9004	116.0302	8	D-net
BYD465	Arthropoda	Insecta	Odonata	Libellulidae	Sympetrinae	/	*Crocothemis*	*Crocothemis servilia*	Larva	Xiuru Xiao	07-Aug-2024	China	Hebei	Xiong`an New Area	38.9170	115.8200	8	D-net
BYD373	Arthropoda	Insecta	Odonata	Libellulidae	Sympetrinae	/	*Crocothemis*	*Crocothemis servilia*	Larva	Xiaolong Lin	14-Apr-2024	China	Hebei	Xiong`an New Area	38.7650	116.0200	6	D-net
BYD9	Arthropoda	Insecta	Odonata	Libellulidae	Sympetrinae	/	*Deielia*	*Deielia phaon*	Larva	Xiaolong Lin	16-Jan-2024	China	Hebei	Xiong`an New Area	38.9190	116.0090	6	D-net
BYD358	Arthropoda	Insecta	Odonata	Libellulidae	Sympetrinae	/	*Deielia*	*Deielia phaon*	Larva	Xiaolong Lin	13-Apr-2024	China	Hebei	Xiong`an New Area	38.8850	115.8190	6	D-net
BYD333	Arthropoda	Insecta	Odonata	Libellulidae	Sympetrinae	/	*Deielia*	*Deielia phaon*	Larva	Xiaolong Lin	13-Apr-2024	China	Hebei	Xiong`an New Area	38.9190	116.0090	6	D-net
BYD302	Arthropoda	Insecta	Odonata	Libellulidae	Sympetrinae	/	*Deielia*	*Deielia phaon*	Larva	Xiaolong Lin	17-Apr-2024	China	Hebei	Xiong`an New Area	38.8230	115.8720	6	D-net
BYD222	Arthropoda	Insecta	Odonata	Libellulidae	Sympetrinae	/	*Deielia*	*Deielia phaon*	Larva	Xiaolong Lin	18-Apr-2024	China	Hebei	Xiong`an New Area	38.9040	115.9460	6	D-net
BYD212	Arthropoda	Insecta	Odonata	Libellulidae	Sympetrinae	/	*Deielia*	*Deielia phaon*	Larva	Xiaolong Lin	13-Apr-2024	China	Hebei	Xiong`an New Area	38.9490	115.9610	6	D-net
BYD183	Arthropoda	Insecta	Odonata	Libellulidae	Sympetrinae	/	*Deielia*	*Deielia phaon*	Larva	Mengyu Lv	01-Nov-2023	China	Hebei	Xiong`an New Area	38.9040	115.9510	6	D-net
BYD117	Arthropoda	Insecta	Odonata	Libellulidae	Sympetrinae	/	*Deielia*	*Deielia phaon*	Larva	Xiaolong Lin	18-Jan-2024	China	Hebei	Xiong`an New Area	38.8070	115.8440	6	D-net
BYD446	Arthropoda	Insecta	Odonata	Macromiidae	Macromiinae	/	*Epophthalmia*	*Epophthalmia elegans*	Larva	Xiuru Xiao	02-Aug-2024	China	Hebei	Xiong`an New Area	38.9030	115.7910	7	D-net
BYD365	Arthropoda	Insecta	Odonata	Macromiidae	Macromiinae	/	*Epophthalmia*	*Epophthalmia elegans*	Larva	Xiaolong Lin	14-Apr-2024	China	Hebei	Xiong`an New Area	38.7770	115.8560	6	D-net
BYD568	Arthropoda	Insecta	Odonata	Coenagrionidae	Ischnurinae	/	*Ischnura*	*Ischnura elegans*	Larva	Yuan Yao	06-Nov-2024	China	Hebei	Xiong`an New Area	38.9455	115.9988	7	D-net
BYD485	Arthropoda	Insecta	Odonata	Coenagrionidae	Ischnurinae	/	*Ischnura*	*Ischnura elegans*	Larva	Xiuru Xiao	06-Aug-2024	China	Hebei	Xiong`an New Area	38.8190	115.8520	13	D-net
BYD274	Arthropoda	Insecta	Odonata	Coenagrionidae	Ischnurinae	/	*Ischnura*	*Ischnura elegans*	Larva	Xiaolong Lin	15-Apr-2024	China	Hebei	Xiong`an New Area	38.9030	115.7910	6	D-net

BYD184	Arthropoda	Insecta	Odonata	Coenagrionidae	Ischnurinae	/	*Ischnura*	*Ischnura elegans*	Larva	Mengyu Lv	01-Nov-2023	China	Hebei	Xiong`an New Area	38.8800	115.7550	6	D-net
BYD116	Arthropoda	Insecta	Odonata	Libellulidae	Libellulinae	/	*Orthetrum*	*Orthetrum albistylum*	Larva	Xiaolong Lin	18-Jan-2024	China	Hebei	Xiong`an New Area	38.7750	115.8620	6	D-net
BYD772	Arthropoda	Insecta	Odonata	Coenagrionidae	Coenagrioninae	/	*Paracercion*	*Paracercion calamorum*	Larva	Yuan Yao	08-May-2025	China	Hebei	Xiong`an New Area	38.9441	116.0151	9	D-net
BYD692	Arthropoda	Insecta	Odonata	Coenagrionidae	Coenagrioninae	/	*Paracercion*	*Paracercion calamorum*	Larva	Yuan Yao	06-Nov-2024	China	Hebei	Xiong`an New Area	38.7765	115.8555	7	D-net
BYD610	Arthropoda	Insecta	Odonata	Coenagrionidae	Coenagrioninae	/	*Paracercion*	*Paracercion calamorum*	Larva	Yuan Yao	06-Nov-2024	China	Hebei	Xiong`an New Area	38.9444	116.0148	8	D-net
BYD482	Arthropoda	Insecta	Odonata	Coenagrionidae	Coenagrioninae	/	*Paracercion*	*Paracercion plagiosum*	Larva	Xiuru Xiao	06-Aug-2024	China	Hebei	Xiong`an New Area	38.8400	115.9510	5	D-net
BYD92	Arthropoda	Insecta	Odonata	Coenagrionidae	Coenagrioninae	/	*Paracercion*	*Paracercion* sp. 1XA	Larva	Xiaolong Lin	19-Jan-2024	China	Hebei	Xiong`an New Area	38.8440	115.9900	6	D-net
BYD894	Arthropoda	Insecta	Odonata	Coenagrionidae	Coenagrioninae	/	*Paracercion*	*Paracercion* sp. 1XA	Larva	Yuan Yao	13-May-2025	China	Hebei	Xiong`an New Area	38.8376	115.9505	7	D-net
BYD75	Arthropoda	Insecta	Odonata	Coenagrionidae	Coenagrioninae	/	*Paracercion*	*Paracercion* sp. 1XA	Larva	Xiaolong Lin	18-Jan-2024	China	Hebei	Xiong`an New Area	38.9980	116.0220	6	D-net
BYD742	Arthropoda	Insecta	Odonata	Coenagrionidae	Coenagrioninae	/	*Paracercion*	*Paracercion* sp. 1XA	Larva	Yuan Yao	07-May-2025	China	Hebei	Xiong`an New Area	38.9719	115.9803	7	D-net
BYD68	Arthropoda	Insecta	Odonata	Coenagrionidae	Coenagrioninae	/	*Paracercion*	*Paracercion* sp. 1XA	Larva	Xiaolong Lin	16-Jan-2024	China	Hebei	Xiong`an New Area	38.9980	116.0220	6	D-net
BYD532	Arthropoda	Insecta	Odonata	Coenagrionidae	Coenagrioninae	/	*Paracercion*	*Paracercion* sp. 1XA	Larva	Yuan Yao	06-Nov-2024	China	Hebei	Xiong`an New Area	38.9633	116.0063	6	D-net
BYD51	Arthropoda	Insecta	Odonata	Coenagrionidae	Coenagrioninae	/	*Paracercion*	*Paracercion* sp. 1XA	Larva	Xiaolong Lin	20-Jan-2024	China	Hebei	Xiong`an New Area	38.9440	116.0150	6	D-net
BYD470	Arthropoda	Insecta	Odonata	Coenagrionidae	Coenagrioninae	/	*Paracercion*	*Paracercion* sp. 1XA	Larva	Xiuru Xiao	07-Aug-2024	China	Hebei	Xiong`an New Area	38.9200	115.8860	5	D-net
BYD456	Arthropoda	Insecta	Odonata	Coenagrionidae	Coenagrioninae	/	*Paracercion*	*Paracercion* sp. 1XA	Larva	Xiuru Xiao	07-Aug-2024	China	Hebei	Xiong`an New Area	38.9190	115.8430	4	D-net
BYD429	Arthropoda	Insecta	Odonata	Coenagrionidae	Coenagrioninae	/	*Paracercion*	*Paracercion* sp. 1XA	Larva	Xiuru Xiao	01-Aug-2024	China	Hebei	Xiong`an New Area	38.9440	116.0150	8	D-net
BYD335	Arthropoda	Insecta	Odonata	Coenagrionidae	Coenagrioninae	/	*Paracercion*	*Paracercion* sp. 1XA	Larva	Xiaolong Lin	13-Apr-2024	China	Hebei	Xiong`an New Area	38.9190	116.0090	6	D-net
BYD273	Arthropoda	Insecta	Odonata	Coenagrionidae	Coenagrioninae	/	*Paracercion*	*Paracercion* sp. 1XA	Larva	Xiaolong Lin	15-Apr-2024	China	Hebei	Xiong`an New Area	38.9030	115.7910	6	D-net
BYD256	Arthropoda	Insecta	Odonata	Coenagrionidae	Coenagrioninae	/	*Paracercion*	*Paracercion* sp. 1XA	Larva	Xiaolong Lin	14-Apr-2024	China	Hebei	Xiong`an New Area	38.9440	116.0150	6	D-net
BYD225	Arthropoda	Insecta	Odonata	Coenagrionidae	Coenagrioninae	/	*Paracercion*	*Paracercion* sp. 1XA	Larva	Xiaolong Lin	15-Apr-2024	China	Hebei	Xiong`an New Area	38.9460	115.9990	6	D-net
BYD372	Arthropoda	Insecta	Odonata	Coenagrionidae	Coenagrioninae	/	*Paracercion*	*Paracercion calamorum*	Larva	Xiaolong Lin	14-Apr-2024	China	Hebei	Xiong`an New Area	38.7650	116.0200	6	D-net
BYD369	Arthropoda	Insecta	Odonata	Coenagrionidae	Coenagrioninae	/	*Paracercion*	*Paracercion calamorum*	Larva	Xiaolong Lin	14-Apr-2024	China	Hebei	Xiong`an New Area	38.7650	116.0200	6	D-net
BYD170	Arthropoda	Insecta	Odonata	Coenagrionidae	Coenagrioninae	/	*Paracercion*	*Paracercion calamorum*	Larva	Mengyu Lv	01-Nov-2023	China	Hebei	Xiong`an New Area	38.7480	116.0350	6	D-net
BYD166	Arthropoda	Insecta	Odonata	Coenagrionidae	Coenagrioninae	/	*Paracercion*	*Paracercion calamorum*	Larva	Mengyu Lv	01-Nov-2023	China	Hebei	Xiong`an New Area	38.7690	116.0080	6	D-net
BYD105	Arthropoda	Insecta	Odonata	Coenagrionidae	Coenagrioninae	/	*Paracercion*	*Paracercion calamorum*	Larva	Xiaolong Lin	18-Jan-2024	China	Hebei	Xiong`an New Area	38.9160	115.7430	6	D-net
BYD942	Arthropoda	Insecta	Odonata	Platycnemididae	Platycnemidinae	/	*Platycnemis*	*Platycnemis phyllopoda*	Larva	Yuan Yao	15-May-2025	China	Hebei	Xiong`an New Area	38.9035	115.7929	10	D-net
BYD519	Arthropoda	Insecta	Odonata	Platycnemididae	Platycnemidinae	/	*Platycnemis*	*Platycnemis phyllopoda*	Larva	Xiuru Xiao	02-Aug-2024	China	Hebei	Xiong`an New Area	38.7650	116.0200	7	D-net
BYD357	Arthropoda	Insecta	Odonata	Platycnemididae	Platycnemidinae	/	*Platycnemis*	*Platycnemis phyllopoda*	Larva	Xiaolong Lin	13-Apr-2024	China	Hebei	Xiong`an New Area	38.8850	115.8190	6	D-net
BYD936	Arthropoda	Insecta	Odonata	Platycnemididae	Platycnemidinae	/	*Pseudocopera*	*Pseudocopera ciliata*	Larva	Yuan Yao	15-May-2025	China	Hebei	Xiong`an New Area	38.9035	115.7929	10	D-net
BYD366	Arthropoda	Insecta	Odonata	Platycnemididae	Platycnemidinae	/	*Pseudocopera*	*Pseudocopera ciliata*	Larva	Xiaolong Lin	14-Apr-2024	China	Hebei	Xiong`an New Area	38.7770	115.8560	6	D-net
BYD258	Arthropoda	Insecta	Odonata	Libellulidae	Trithemistinae	/	*Pseudothemis*	*Pseudothemis zonata*	Larva	Xiaolong Lin	14-Apr-2024	China	Hebei	Xiong`an New Area	38.9440	116.0150	6	D-net
BYD81	Arthropoda	Insecta	Odonata	Gomphidae	Lindeniinae	/	*Sinictinogomphus*	*Sinictinogomphus clavatus*	Larva	Xiaolong Lin	18-Jan-2024	China	Hebei	Xiong`an New Area	38.8860	115.9970	6	D-net
BYD509	Arthropoda	Insecta	Odonata	Gomphidae	Lindeniinae	/	*Sinictinogomphus*	*Sinictinogomphus clavatus*	Larva	Xiuru Xiao	04-Aug-2024	China	Hebei	Xiong`an New Area	38.8870	116.0960	10	D-net
BYD508	Arthropoda	Insecta	Odonata	Gomphidae	Lindeniinae	/	*Sinictinogomphus*	*Sinictinogomphus clavatus*	Larva	Xiuru Xiao	04-Aug-2024	China	Hebei	Xiong`an New Area	38.8870	116.0960	10	D-net
BYD349	Arthropoda	Insecta	Trichoptera	Hydropsychidae	Hydropsychinae	/	*Cheumatopsyche*	*Cheumatopsyche brevilineata*	Larva	Xiaolong Lin	13-Apr-2024	China	Hebei	Xiong`an New Area	38.9160	115.7430	6	D-net
BYD348	Arthropoda	Insecta	Trichoptera	Hydropsychidae	Hydropsychinae	/	*Cheumatopsyche*	*Cheumatopsyche brevilineata*	Larva	Xiaolong Lin	13-Apr-2024	China	Hebei	Xiong`an New Area	38.9160	115.7430	6	D-net
BYD64	Arthropoda	Insecta	Trichoptera	Ecnomidae	/	/	*Ecnomus*	*Ecnomus tenellus*	Larva	Xiaolong Lin	16-Jan-2024	China	Hebei	Xiong`an New Area	38.9980	116.0220	6	D-net
BYD505	Arthropoda	Insecta	Trichoptera	Ecnomidae	/	/	*Ecnomus*	*Ecnomus tenellus*	Larva	Xiuru Xiao	04-Aug-2024	China	Hebei	Xiong`an New Area	38.8980	116.0950	7	D-net
BYD417	Arthropoda	Insecta	Trichoptera	Ecnomidae	/	/	*Ecnomus*	*Ecnomus tenellus*	Larva	Xiuru Xiao	31-Jul-2024	China	Hebei	Xiong`an New Area	38.9860	115.9700	10	D-net
BYD571	Arthropoda	Insecta	Trichoptera	Leptoceridae	Leptocerinae	/	*Leptocerus*	*Leptocerus biwae*	Larva	Yuan Yao	06-Nov-2024	China	Hebei	Xiong`an New Area	38.9455	115.9988	7	D-net
